# Leaftronics: Natural lignocellulose scaffolds for sustainable electronics

**DOI:** 10.1126/sciadv.adq3276

**Published:** 2024-11-08

**Authors:** Rakesh R. Nair, Jakob Wolansky, Kai Uhlig, Ali Solgi, Laura Teuerle, Tianyi Zhang, Jonas Schröder, Tobias Antrack, Johannes Benduhn, Hans Kleemann, Karl Leo

**Affiliations:** ^1^ Dresden Integrated Center for Applied Physics and Photonic Materials (IAPP) and Institute for Applied Physics, Technische Universität Dresden, 01187 Dresden, Germany.; ^2^ Leibniz-Institut für Polymerforschung Dresden e.V., 01069 Dresden, Germany.

## Abstract

The global rise in electronic waste is alarming, driven by the persistent use of glass, epoxy, and plastic substrates owing to their cost, stability, flexibility, and transparency. This underscores the need for biodegradable alternatives with similar properties. This study shows that leaf-derived lignocellulose scaffolds can stabilize bio-sourced, solution-processed polymers by acting as natural sequestering media. Such reinforced films, even when based on gelatin (*T*_g_ ~ 60°C), can endure processes over 200°C. We demonstrate dip-coated ethyl cellulose films for commercially viable reflow soldered circuitry. The films offer high flexibility, more than 80% transparency, and surface roughness below 5.5 nm. Advanced OPDs and OECTs fabricated on these films perform comparably to those on glass and the low material cost and simple fabrication process yields a minimal carbon footprint of 1.6 kgCO_2_/m^2^. This work thus opens a vista of possibilities for biodegradable polymers heretofore considered unsuitable for making temperature-stable substrates for state-of-the-art electronics applications.

## Teaser

Quasi-fractal leaf scaffolds can thermally stabilize biodegradable polymer films for use as substrates in advanced electronics.

## INTRODUCTION

The global accumulation of electronic waste now exceeds 50 million metric tons per year (*1*) and is projected to double within the next 20 to 30 years (*1*). The substrate onto which electronic components are soldered may itself contribute between 20 and 60% of the total mass of a discarded populated printed circuit board (PCB) (*2*, *3*). These substrates, being nonbiodegradable, constitute a substantial portion of e-waste (electronic waste) generated yearly. In 2019, a mere 17.4% of global e-waste underwent collection and recycling, leaving the remaining 82.6% (44.3 million tons) unaccounted for, with untracked whereabouts (*1*).

Efforts to mitigate this environmental challenge have led to the exploration of various biodegradable substrate materials, as documented in previous reports (*4*, *5*). In contrast to synthetic biodegradable polymers like poly(glycolic acid) and poly(lactic acid), which are relatively expensive and necessitate intricate processing (*6*), biopolymers are naturally occurring and are produced by living organisms. Among these materials, cellulose, abundantly present in nature alongside lignin as a structural unit of land-dwelling flora, stands out as the most prevalent biopolymer on Earth (*7*). Lignin and cellulose (hence “lignocellulose”) also constitute the primary structural components of leaf vasculature (made up of xylem and phloem vesicles). When this vasculature is extracted undamaged from a leaf, it is commonly referred to as a “leaf skeleton” (LS). Although cellulose can naturally form rigid and flexible structures, its properties can be chemically altered to make the resultant material solution processable while retaining biodegradability (*8*, *9*). Such modifications have resulted in inexpensive forms of solution processable bio-derived polymers such as methyl cellulose, ethyl cellulose (EC), and cellulose acetate (*9*, *10*, *11*). However, biodegradable materials often suffer from low thermal stability because the low crystallinity/amorphous nature of the molecular structure that grants the biodegradability (by allowing easy ingress of microbial enzymes) (*12*) also causes the material to easily succumb to elevated temperatures (*13*). Thermomechanical modification of biodegradable polymers has been demonstrated previously via cross-linking or complexing with other synthetic materials (*14*, *15*). However, developing a cost-effective method to produce thermally stable films of low glass transition temperature (*T*_g_) biodegradable materials without complex chemical modifications while preserving their inherent flexibility, biodegradability, and transparency would open new research avenues, particularly in thin-film and commercial thick-film electronics.

**Fig. 1.**
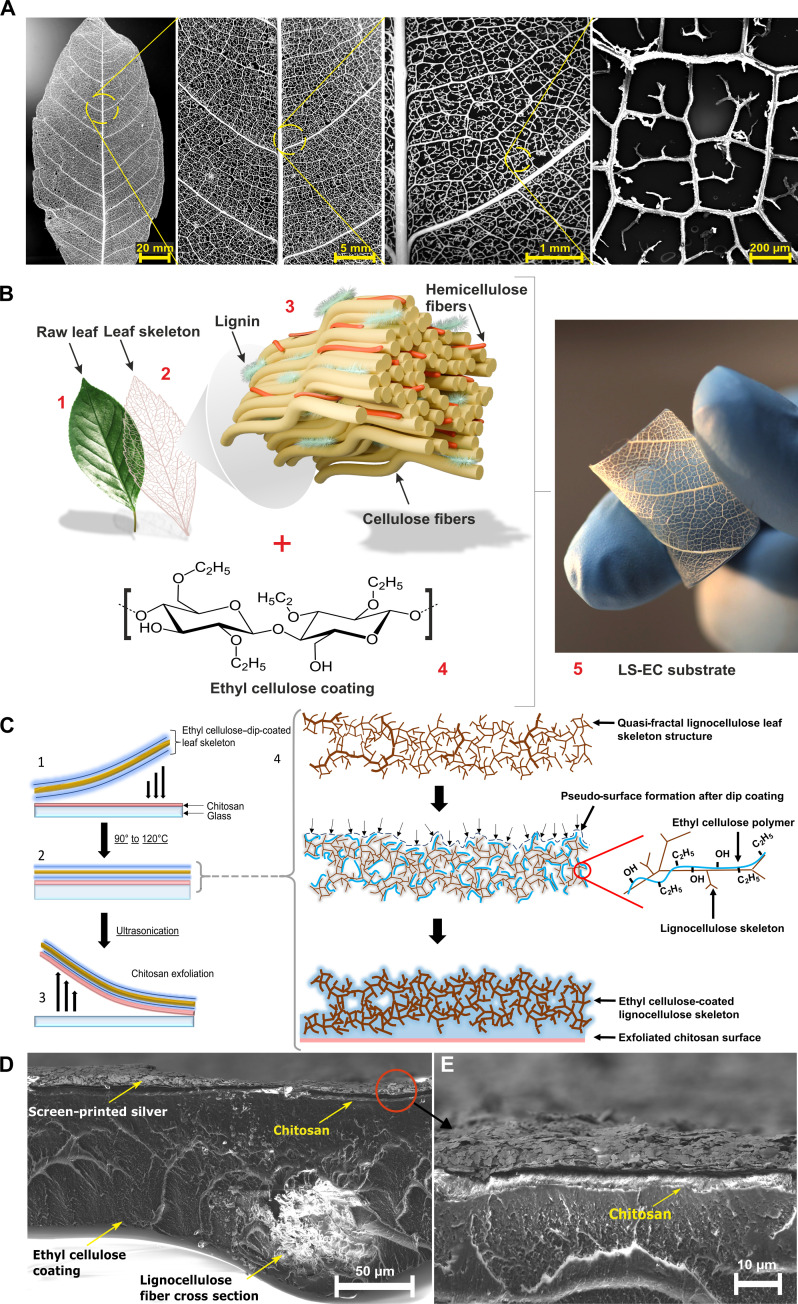
Lignocellulose quasi-fractals and their coating. (**A**) Magnolia LS quasi-fractal structure at different magnifications. Scale bars from left to right: 20 mm, 5 mm, 1 mm, and 200 μm [scanning electron microscopy (SEM) image]. (**B**) Fabrication process of Leaftronic substrates, showing (1) raw leaf; (2) the extracted lignocellulose scaffold (LS); (3) microscopic illustration of cellulose microfibrils bonded together with lignin and supported by hemicellulose; (4) ethyl cellulose (EC) molecular structure, which, in the solution processed form, is coated onto the extracted lignocellulose scaffold; and (5) image of the final LS-EC substrate. (**C**) Chitosan (CS) exfoliation process for providing a layer of CS on top of LS-EC: (1) dip-coated scaffold placed on to a CS-coated borosilicate glass slide, which is (2) dried at 90° to 120°C followed by (3) exfoliation and peel-off. Illustration of the hypothesized substrate formation process, showing freshly extracted LS (top) being dip coated in solution processed EC (middle) (the arrows indicate the EC polymer filling up the pores of the leaf scaffold and creating a film due to surface tension as it moves downward under gravity), which, after drying and glass exfoliation (bottom), results in an LS-EC-CS substrate with a smooth, flat side. (**D**) SEM cross section of an LS-EC-CS substrate with screen-printed Ag (scale bar, 50 μm), and (**E**) the close-up image of the interface (right; scale bar, 10 μm).

Therefore, in an effort to create a low-cost, biodegradable substrate capable of catering to the needs of both commercial and thin-film electronics, we propose LS-reinforced electronics (Leaftronics) as a novel, environmentally friendly platform for electronic devices and circuitry (Fig. 1). The quasi-fractal structure of leaf derived lignocellulose scaffolds functions as a film-forming mesh that is well suited for the uptake and subsequent coalescing of solution processed organic materials to form freestanding sheets. This suffusion of solution processed materials can be achieved via simple dip coating as is elaborated upon in further sections. We quantitatively demonstrate the efficacy of Leaftronics by choosing a generic polymer such as EC and upgrading its properties as a quasi-transparent, flexible, and biodegradable substrate with an average surface roughness of 3.7 nm. We demonstrate microcontroller-based reflow soldered circuitry and physical vapor deposited (PVD) optoelectronic devices along with printed organic electrochemical transistors (OECTs) and PCB traces on these substrates.

We additionally study the sustainability parameters of this substrate and calculate the CO_2_ footprint generated from fabricating such a film in the latter part of this publication. Additional to the sustainability parameters, we also consider whether the substrates fit within the tenets of a circular economic system. The concept of a circular economy was arguably first introduced in 1990 (*16*, *17*) and refers to an economic model focusing on the minimization of waste by deliberate design of products for reuse via upcycling or recycling. This results in a closed-loop system where products are mass produced with the intent of eventual material recovery and reuse. Therefore, this model moves away from the current global approach of production, consumption, and subsequent waste generation. We implement such circular economic principles in this research work by developing a method of tuning the substrate surface properties for reflow soldered electronics such that soldered components can be easily extracted from the substrate at the end of life (EoL) of the circuit board. The developed method allows not only the nondestructive recovery of electronic components but also the metal used to print circuit interconnects without damaging the substrate itself.

We also follow this up by developing a preliminary energy and material flow diagram of this process as a starting point for future inventory analysis tasks during life cycle assessments (LCAs) of Leaftronic applications (fig. S18) (*18*). LCA is an ISO-standardized method to quantify and qualify the environmental impact of products and processes. The ISO standards 14040 and 14044 provide a framework for the analysis itself as well as the inventory data. The analysis comprises four distinct steps, which are executed sequentially with the possibility of any step being revisited and reevaluated at any point to enhance the LCA results. The process involves the following:

1) Goal and scope definition: The objective of the analysis is established, and the functional unit is specified to describe the analyzed “product” in comprehensive detail.

2) Inventory analysis: Involves data collection encompassing the cataloguing of all materials used, their quantities, and the energy consumption of machinery across all fabrication processes.

3) Impact assessment: Data from the inventory analysis are used to calculate the environmental impact with respect to predefined impact categories.

4) Interpretation and conclusion: The results are interpreted, conclusions are drawn, and the analysis is revisited to identify potential improvements in the methods or inventory.

Overall, we demonstrate a facile method to potentially adapt the entire class of myriad low *T*_g_, biodegradable materials for high-temperature processing by showing that the key might lie in reinforcing such materials with quasi-fractal scaffolds. We show that suitable biodegradable scaffolds can be readily harnessed directly from natural leaves and that the resulting reinforced substrates might have the potential to actively resist deliquescing under temperatures even above 200°C without needing complex chemical stabilization while also retaining their biodegradability. It is believed that further research into this discovery, especially considering techniques of cross-linking such bio-based materials with synthetic ones (*14*, *19*), along with novel techniques of directly modifying plant vascular tissue via in vivo polymerization (*20*) may have the potential to sustainably introduce nature-inspired resilience into modern manufacturing.

## RESULTS

**Fig. 2.**
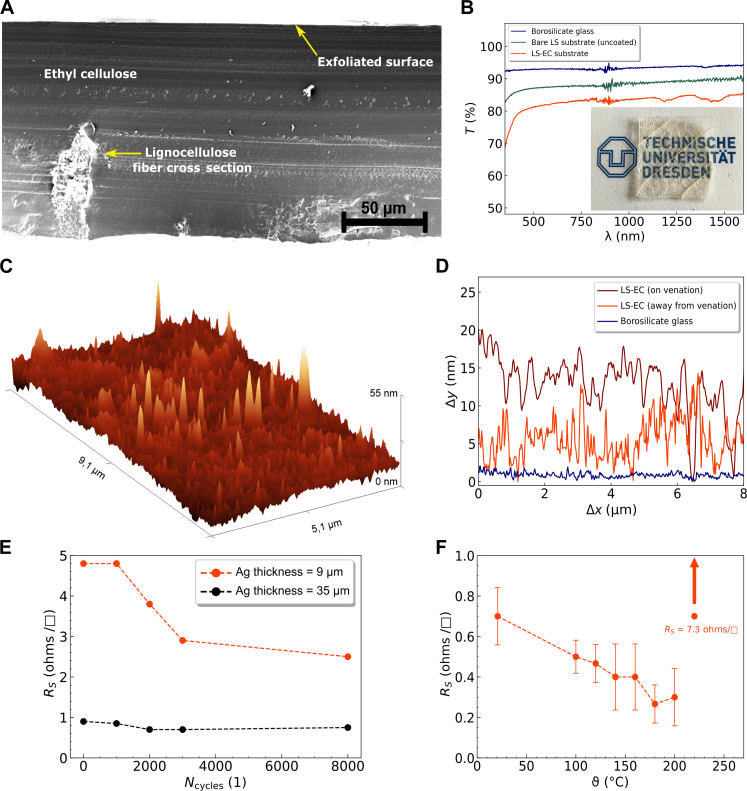
Leaftronic substrate properties. (**A**) SEM image of the LS-EC substrate cross section showing the exfoliated surface (top) being smoother than the opposite side. (**B**) Optical transmittance measurement of bare glass (blue); uncoated, freshly extracted leaf-skeleton (green); and LS-EC substrate (red) (inset: demonstration of substrate transparency with respect to a printed image). (**C**) Atomic force microscopy (AFM) profile of the LS-EC substrate surface; root mean square (RMS), ~3.3 nm. (**D**) Comparison of the surface profile of the LS-EC substrate when AFM is performed on a location directly over the embedded venation (brown, top) (RMS roughness, ~5.4 nm), away from the venation (red, middle) (RMS roughness, ~3.3 nm), compared to the surface of standard borosilicate glass substrates (blue, bottom) (RMS roughness, ~0.8 nm). (**E**) Variation in sheet resistance (*R*_s_) of screen-printed Ag layers (2 cm by 2 cm) through 8000 bending cycles (165-31 screen gave a layer thickness of ~9 μm, and 61-64 screen gave a layer thickness of ~35 μm). (**F**) Variation in sheet resistance (*R*_s_) of screen-printed Ag layers (61-64 screen) with respect to temperature, sheet resistance crosses 7 ohms/□ beyond 230°C and is indicated with an arrow because it exceeds the *y*-axis range.

Although LSs of myriad species of tree leaves can be purchased commercially, we tested the feasibility of Leaftronics by directly extracting LSs from the leaves of locally growing magnolia trees. This also results in the avoidance of extraneous carbon emissions resulting from processing, packaging, and shipping of commercially sold LSs and thus results in a more realistic eventual estimation of the carbon footprint. LSs are the lignocellulose venations present within leaves and are shown at different scales of magnification in Fig. 1A for a magnolia leaf used in this work. These venations are extracted intact from the green mesophyll of a fresh leaf via alkaline removal of the leaf biomass in a heated aqueous solution of sodium carbonate decahydrate (Na_2_CO_3_·10H_2_O) (Fig. 1B, 1 and 2) (details in Experimental design).

The skeletons subsequently undergo dip coating in EC dissolved in 2-butoxyethanol (fig. S1). The EC (Fig. 1B.4) fills the porous, quasi-fractal geometry of the LS (Fig. 1A), which is primarily made up of cellulose, hemicellulose, and lignin (Fig. 1B.3), resulting in a functional, flexible surface (Fig. 1B.5).

We chose EC (Fig. 1B.4) because it is among the few known biodegradable polymers with a relatively high *T*_g_ (*21*), which already provides it with notable thermal resistance. EC is also hydrophobic, insoluble in water, and stable in pH environments ranging from 3 to 11 while being benign in terms of handling since it is classified as a food-grade material (*22*, *23*). 2-Butoxyethanol is chosen as a solvent here because of its low vapor pressure, easy biodegradability, and low toxicity (approved by the US Food and Drug Administration as a food additive) (*24*). This combination is a consequence of our goal to ensure that the substrates remain sustainable while being modified for high-temperature processing. Therefore, the combination of a purely biological and a bio-sourced material (the latter dissolved in a biodegradable solvent) results in an environmentally benign substrate (Fig. 1B.5) with emergent properties gained from the fractal frame provided by the lignocellulose network and the tensile strength provided by the EC chains. This not only forms an excellent flexible substrate for device fabrication but also, by being biodegradable, conforms to circular economic principles.

### Surface modifications

One of the reasons for which substrates in organic electronics need surface treatment is due to the poor adhesion of water-based printing inks, especially on hydrophobic materials like EC. A coating of the biodegradable, biologically derived cationic polymer, chitosan (CS), over the LS-EC (LS-reinforced EC) substrate mitigates the issue in a facile manner. The hydrophilic nature of CS and its ability to chelate metals (*25*) results in excellent adhesion of water-based PEDOT:PSS and Ag inks on the substrate, now referred to as LS-EC-CS (fig. S2, A to C).

Another notable substrate-related issue in organic electronics is the surface roughness, especially when considering fabrication methods like PVD. We developed a facile glass exfoliation technique (Fig. 1C, 1 to 3) (details in Experimental design) to optimize the substrate surface morphology with and without CS. Figure 1C.4 illustrates the dip-coating process with respect to the interaction between the LS and EC polymer chains [both being cellulosic adhere well, and Fig. 2A shows a scanning electron microscopy (SEM) cross section of an LS fiber thoroughly coated with EC]. The surface tension of the EC solution creates a pseudo-surface, surrounding the entirety of the LS structure immediately following dip coating. When this EC suffused LS is allowed to dry on a glass substrate containing a coating of CS (~10 to 20 μm), the negatively charged EC adheres well with the cationic CS (*26*, *27*) and reliably pulls away this CS surface during exfoliation (Fig. 1D). This results in the film surface (having conformed to the glass’s surface topology), achieving a high level of smoothness (fig. S4).

This exfoliation technique (to achieve average surface roughness below 5 nm) is also optimized without a CS layer to allow applications where CS is not pertinent. SEM cross section of the LS-EC substrate (Fig. 2A) clearly shows the EC surface’s root mean square (RMS) roughness of 3.7 nm [atomic force microscopy (AFM) measurements in Fig. 2C]. Such low surface roughness was found suitable for consistent deposition of thin (<100 nm) layers of metals or semiconductors using PVD and will be elaborated upon in upcoming sections. AFM measurements also demonstrate that the surface roughness increases to a maximum of ~5.4 nm when measured directly on top of the coated vein structures as shown in fig. S4. The surface roughness of the LS-EC substrate away from the embedded venation and directly on top of the embedded venation is shown in Fig. 2D, along with the roughness of a borosilicate glass substrate as comparison.

### Optical, mechanical, and thermal properties

The exfoliation technique yields a substrate with more than 80% optical transmittance (Fig. 2B). Implementation of thin-film metal deposition through PVD on this substrate enables transparent electrode functionalities (*28*), and the results comparable with that of state-of-the-art transparent conductive electrodes, are provided in fig. S6 and its description.

We also gauged substrate performance by screen printing Ag (SEM cross section shown in fig. S11) and measuring the variation of sheet resistance with respect to increasing temperature and mechanical bending. The substrates show excellent stability against bending and the screen-printed Ag layer remains undamaged even after 8000 bending and relaxation cycles over a 1-cm-diameter roller (Fig. 2E) (details in Experimental design) with a strain of ~3%. The sheet resistance of the screen-printed Ag layer drops with repetitive bending, and we attribute this to the previously reported phenomenon of densification and realignment (*29*, *30*, *31*). “Densification” is the process where randomly oriented nano/microparticles making up a layer are pushed closer together because of the compressive stress experienced during the process of substrate bending, which improves electrical conduction. “Realignment,” on the other hand, is the phenomenon where recurrent bending and relaxation of the substrate allows the nano/microparticles (or the separate sintered domains of the same) to move into better alignment with each other, thus reducing vacant spaces within the layer and improving electrical conduction.

Upon heating, an initial sintering of Ag occurs, which reduces the sheet resistance. The conductivity of the screen-printed layer remains stable afterward (Fig. 2F), despite the softening of the EC underneath that has a *T*_g_ between 130° and 155°C (*32*, *33*). Unlike a substrate made purely from EC, which loses consistency and begins to flow, effectively degrading the printed layers above (see example in fig. S5), the substrates fabricated with the embedded quasi-fractal lignocellulose scaffolds are coalesced into place even when liquefied under heat. This results in the substrate maintaining consistency even at temperatures far exceeding the *T*_g_ of EC. Upon cooling on a nonsticky surface like parchment paper, the substrate coalesces back to its stable form as it is drawn in by the lignocellulose scaffold (fig. S7). This emergent property allows these substrates to function at temperatures above EC’s *T*_g_, facilitating reflow soldering profiles exceeding 210°C peaks for commercially equivalent circuit fabrication which generally uses 160° to 180°C temperatures.

To prove that this emergent stability is conferred by the embedded lignocellulose scaffold, we also checked whether such Leaftronic modifications would enhance the properties of polymers with far lower temperature stability. Gelatin liquefies completely by 60°C from a gel-like state at room temperature and is, therefore, unfit as an unmodified substrate for processes requiring elevated temperatures. Comparing gelatin films (plasticized with 3% glycerol) with and without scaffold reinforcement (fig. S8) shows that reinforced gelatin films retain their shape even when heated up to 225°C, while films without reinforcement expectedly melt and lose their structure below 100°C. Previous reports have shown that gelatin films begin active degradation close to 200°C due to the complete loss of water from within the film and the excess mobility of individual polymer chains, a phenomenon called supercontraction (*34*, *35*). Although thermal decomposition, being a material property, is inevitable (as evidenced by bubbles in the film at 224°C), the film structure, however, is stably retained, especially when supercontraction is known to reduce the lateral film dimensions by as much as 30% (*34*).

Coming back to the LS-EC substrates, thermomechanical testing (figs. S9 and S10 and table S2) reveals that they occupy an ideal niche between commonly used organic and inorganic substrates (Table 1) while being sustainable in terms of production and raw material sourcing.

**Table 1.** Thermomechanical properties of common flexible substrates.

Material	Elastic modulus (GPa)	Elongation (%)	Transparency (%)	Dielectric constant (Ɛ_r_)	Glass transition temperature (°C)	Thermal expansion (α_L_) (× 10^6^/K)
Synthetic Hydrogels	39 × 10^−6^ to 907 × 10^−6^ (*54*, *55*)	>1000 (*54*, *55*)	> 90 (*56*)	5.0–55 (*57*)	160–>180 (*58*)	_
PDMS	0.4 × 10^−3^ to 5 × 10^−3^ (*54*, *59*)	~93 (*54*, *60*)	~85–90 (*61*)	2.65 (*62*, *63*)	~−123 (*64*)	~212 (*65*)
**Leaftronic substrate** **(LS-EC)(this work)**	**54.02 × 10**^**−3**^ **± 30.92 × 10**^**−3**^	**6.82 ± 2.10**	**~85**	**3.4**	**~95.05 ± 1.22**	**81.8 ± 19.5** **(−20° to 100°C)**
Paper	1.05–4.6 (*66*)	1.0–20 (*67*)	~30 (*68*)	2.0–4.0 (*69*)	~174 (*70*)	3.6–16.2 (*71*)
PET	2.3 ± 0.3 (*54*, *72*)	~12 (*73*)	90.4 (*54*, *68*)	3.5 (*63*, *74*)	78 (*75*)	15 (*76*)
PI	2.5 (*54*, *77*)	~3 (*54*, *78*)	30–60 (*54*)	2.9 (*63*, *79*)	~360 (*75*)	33.4–53.2 (*80*)
PEN	3.3 ± 0.4 (*19*, *81*)	~7(*73*)	87.0 (*82*)	2.9 (*63*)	120 (*75*)	18–20 (*75*)

In terms of sustainable substrates, paper is often proposed as the flexible and biodegradable material of choice for organic and hybrid electronics (*36*–*38*). However, paper requires a significant amount of processing and energy expenditure: The pulp-and-paper industry is one of the world’s top five polluters, using more water per ton of material than any other industry while generating about the same mass in greenhouse gas emissions (*39*). The substrates presented in this study demand significantly reduced processing (table S3), eliminate the use of toxic or carcinogenic chemicals, and demonstrate a comparable dielectric constant as paper. They are also based on an abundant, naturally occurring biopolymer scaffold, which is further enhanced with minimal amounts of bio-sourced polymeric coatings derived from the two most abundant biomaterials on Earth: cellulose and chitin (*40*). These substrates also offer transparency without being as expensive or process intensive as transparent nano-fibrillated paper (*36*) as measurements listed in Table 1 show. In addition, a higher modulus of elasticity and lower thermal expansion than that of polydimethylsiloxane (PDMS) is observed. When coupled with a thermal competence toward reflow soldering (elaborated upon in the next section), Leaftronic PCBs gain a strategic edge over PDMS, polyethylene terephthalate, and polyimide as a flexible PCB material in specific applications as tabulated in table S1.

### PCB fabrication

**Fig. 3.**
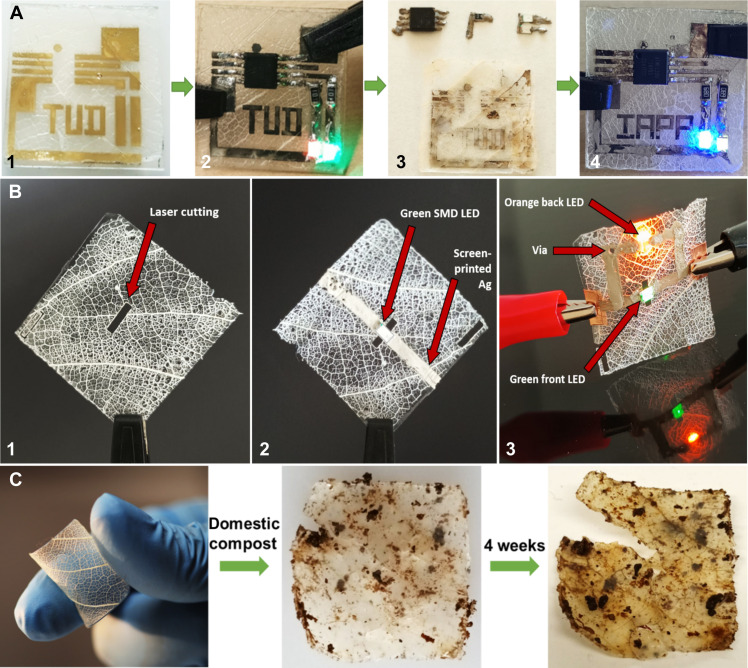
Biodegradable PCBs for commercial reflow soldered electronics. (**A**) Demonstration of PCB application on the LS-EC-CS substrates (2.5 cm by 2.5 cm) showing (1) inkjet-printed Ag tracks designed for an SOIC8 chip implementing additive manufacturing; (2) a functioning, in-circuit programmed, MCU-based, reflow soldered circuit on a LS-EC-CS substrate, controlling two LEDs via pulse width modulation (PWM); (3) extracted components after treatment with diluted acetic acid (0.4%) at room temperature; and (4) same components (except for the resistors due to being fused with solder) reused on a fresh substrate (movie S1). (**B**) Laser structuring performed on LS-EC substrates showing (1) a 0.5 mm–by–2 mm slot cut using a CO_2_ laser, (2) an SMD LED affixed over the slot with electrodes attached to screen-printed contacts using conducting glue, and (3) Ag tracks connected to the back of the substrate by fabricating a conducting-via by screen printing over a laser cut hole to light up two LEDs simultaneously with power supplied only to one side. (**C**) Leaf substrate in compost (1) after a week (2) and after 4 weeks (3).

Reflow soldering is generally used to fix commercial thick-film inorganic electronic components to etched copper-clad laminated PCBs. Here, we implement functional printing techniques for additive fabrication, making any etching-related pollutive treatments for the fabrication of PCB interconnects redundant (Fig. 3A, 1 and 2). We fabricate circuit traces using both inkjet and screen printing and chose Ag-based inks to ensure high conductivity. The choice of using Ag is also informed by the fact that conductivity can be maintained in such interconnects without requiring excessive protection against oxidation. Figure 3A.1 shows inkjet printed circuit traces deposited onto the LS-EC-CS substrates using commercially available nanoparticle Ag ink. The CS top layer is necessary because the Ag nanoparticle ink is water based and, hence, does not adhere well on LS-EC substrates due to the hydrophobicity of EC (fig. S2B). No such issues were noted with screen printing because the ink used is solvent based and flows well on LS-EC substrates (screen-printed version of the same circuit is shown in fig. S16; SEM images are shown in fig. S11). Screen printing being a contact deposition process also fosters better adhesion by allowing Ag particles to penetrate deeper into the substrate surface (fig. S11A) (adhesion testing is shown in fig. S12 and associated description). The CS layer ensures satisfactory adhesion of the Ag nanoparticle ink via the chelation of Ag (*25*). The significantly improved adhesion of water-based inks on CS-coated substrates as compared to that of the LS-EC substrates is shown in fig. S2C. The SOIC8 chip adheres strongly to the substrate following reflow soldering (contact resistance of ~10 milliohms), thus allowing in-circuit programming of the microcontroller unit (MCU) with commercial test clips directly clamped onto the MCU pins. The superior adhesion of the components is also a result of the CS top layer softening beyond its *T*_g_ (<150°C) and effectively gluing the components in place upon cooling. The digital outputs were programmed to sequentially fade-in and fade-out two surface-mount device (SMD) light-emitting diodes (LEDs) using pulse width modulation (PWM), and an image of the circuit in operation is shown in Fig. 3A.2 (movie S1). Circuit traces fabricated using screen printing showed the same performance; however, the screen-printing ink (being microparticle based) has a lower environmental impact than the Ag nanoparticle ink used in inkjet printing (*41*) and, hence, is used in further experiments as shown in Fig. 3B for example.

Figure 3A.2 shows components’ reflow soldered onto the LS-EC-CS substrate with inkjet-printed Ag interconnects. The soldering temperature profile reaches a maximum of 245°C for 5 s, which is 25°C higher than the temperature at which CS begins to degrade (*42*). Although low-temperature soldering is also used in the industry (130° to 220°C) (*43*), the effect of heating the LS-EC-CS substrate to temperatures higher than 220°C is the partial degradation of CS where the metal interconnects are deposited. This is evident from the discoloration left over after the components and the CS layer have been removed via ultrasonication for 45 min after immersion of the PCB in a 0.4% acetic acid solution Fig. 3A.3.

### PCB machining

PCBs are often realized with components on both sides in two-layer circuit topologies, necessitating the fabrication of conducting vias or insulating air gaps. Because of their softness, holes can be fashioned on these substrates via die cutting, plotting, drilling, or similar facile methods, following this, screen printing a conductive ink over them results in the ink percolating over the walls of these holes, making them conductive (Fig. 3B.3). We also performed experiments with laser cutting for the creation of air gaps (Fig. 3B, 1 and 2) underneath components that are, at times, needed for separating high voltage lines, heat dissipation, or inverted “bottom-emitting” LEDs in PCBs (*44*). Figure 3B.3 shows LEDs lit up on both sides of the substrate from power supplied to one side.

### Biodegradability

Although literature is sparse, studies on cellulose-based polymers show that higher degree of substitution (as is the case with EC) directly leads to improved barrier properties and, thus, a reduced rate of biodegradation (*10*). Within the circular economic model, recycling is understood to be much more beneficial to the environment than biodegradation (*36*). Therefore, a substrate that does not initiate immediate biodegradation upon disposal allows ample time for collection, recovery of materials/components, and potential relocation of the rest to designated composting areas, such as biogas plants. Biodegradation experiments with standard domestic compost are depicted in Fig. 3C. After 4 weeks, the substrate (Fig. 3C, 1 and 2) cannot be handled with forceps without causing severe damage (Fig. 3C.3), indicating the initiation of biodegradation. Because the substrates are made of nontoxic materials and can foster the growth of yeast (*Saccharomyces cerevisiae*) on the surface, biodegradation via fermentation may also be a possibility (fig. S13).

### Recycling

Commercial electronics typically demand elevated temperatures for desoldering components for reuse. The LS-EC-CS substrates, however, permit component extraction at room temperature without incurring damage, making them suitable for reuse in entirely different circuit topologies. This is possible because the components are soldered via reflow soldering onto the silver interconnects, which are printed on the CS top layer of the substrate (fig. S11C). Although CS shows good adhesion with the naturally negatively charged EC, pH environments of 6 and below cause the amino groups in CS to protonate and render it soluble. When the assembled PCB is ultrasonicated in diluted (0.4%) acetic acid solution (CH_3_COOH) for 2 hours, the CS layer dissolves, and the components detach from the substrate (Fig. 3A.3). CS dissolution also ensures the removal of Ag interconnects because CS is a strong chelating agent for Ag. The low acetic acid concentration also does not damage the underlying EC, thus maintaining its smooth surface topology. The extracted components (including the MCU programmed to drive two LEDs) are perfectly functional after recovery and drying, and a fresh circuit built from the recovered MCU and LEDs functioned as before on a new LS-EC-CS substrate without requiring any reprogramming, as shown in Fig. 3A.4 (movie S2).

### Emerging thin-film device technologies

Above, we have shown that this novel substrate technology has notable potential to reduce the carbon footprint of the multibillion-dollar PCB industry. In the following, we show that Leaftronics also opens up new possibilities within the printed and thin-film organic electronics domains by providing a stable, flexible, transparent, and biodegradable alternative to glass and plastic substrates.

**Fig. 4.**
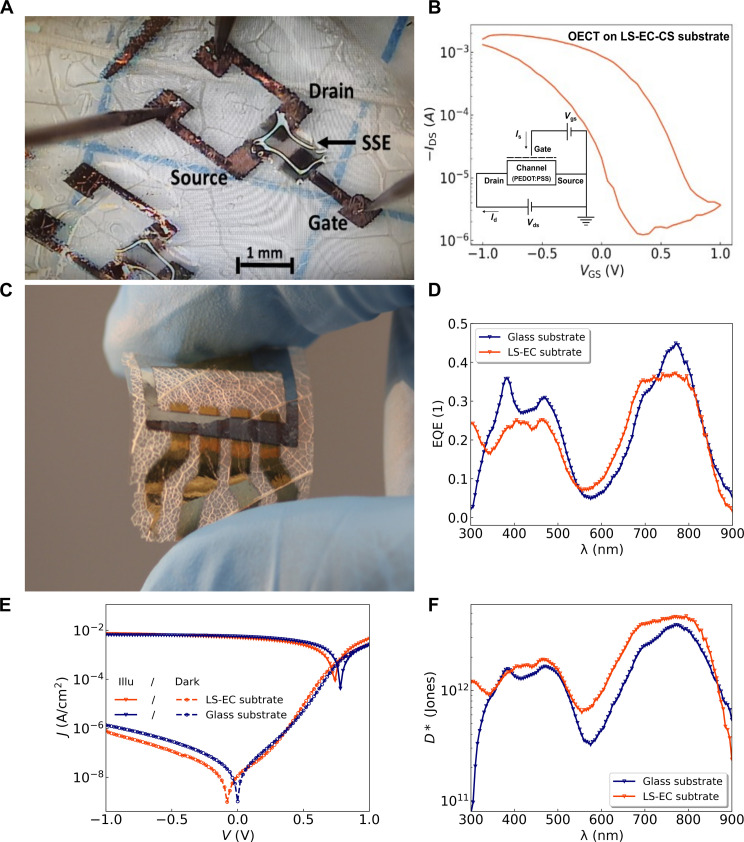
State-of-the-art thin-film device applications. (**A**) Inkjet-printed OECT on LS-EC substrate showing the solid-state electrolyte (SSE). (**B**) Transfer characteristics of the inkjet-printed OECT showing hysteresis. *I*_DS_, *I*_d_, and *I*s: drain-source current; *V*_gs_ and *V*_GS_: gate-source voltage; *V*_ds_: drain-source voltage. (**C**) OPD device on LS-EC substrate. (**D**) EQE spectra. (**E**) J-V characteristics in darkness and under illumination and resulting specific detectivity spectra (**F**) of encapsulated devices on glass and un-encapsulated devices on LS-EC substrates.

Figure 4A shows an LS-EC substrate with an inkjet-printed OECT using a PEDOT:PSS channel and using an ultraviolet (UV) curing solid-state electrolyte (SSE) based on the ionic liquid 1-ethyl-3-methylimidazolium ethyl sulfate (*45*). The SSE, also being inkjet printed, makes the OECT much more practical in terms of fabrication and portability, making it advantageous for applications such as in vivo sensing and neuromorphic computing (*46*). Figure 4B shows the transfer characteristics of the OECT, illustrating the typical hysteretic behavior. The output characteristics and impedance analysis are shown in fig. S14, which, when compared to standard devices on glass, show identical figures of merit [capacitance-mobility product (μC*)] along with good on/off-switching.

Figure 4C shows a pin-type state-of-the-art thin-film organic photodiode (OPD) with a semitransparent top electrode fabricated on the LS-EC substrates (stack information in fig. S15). Figure 4 (D and E) depicts the external quantum efficiency (EQE) spectra and current density–voltage curves for devices on glass (blue) and LS-EC substrates (red), revealing comparable characteristics and resulting in similar open-circuit voltages and photocurrents. Consequently, the thermal noise calculated from the shunt resistance is equal, resulting in a maximum specific detectivity D* of 5 × 10^12^ Jones for the LS-EC sample and 4 × 10^12^ Jones for the sample on glass (Fig. 4F). The good device performance of the OPDs on glass and LS-EC substrates demonstrate efficient charge injection and transportation behavior, thus indicating a well working interface between the bottom electrode and the device stack on top of it. The differences increase over time because of the superior barrier properties of glass not only as an encapsulation material but also as a substrate, when compared to LS-EC as shown in fig. S15C.

### Sustainability aspects of Leaftronics

Table S3 shows the total carbon footprint (TCF) calculations for the Leaftronic substrates fabricated on a lab scale. With a TCF of 1.6 kgCO_2_/m^2^, Leaftronics outperforms the state-of-the-art PCB substrate flame retardant 4 (FR4) by more than one order of magnitude and paper-based PCBs by a factor of 3. We address other important techno-economic properties of our substrates and compare them with FR4 and paper in Table 2.

**Table 2.** Overview table on sustainability-relevant properties of PCB substrate materials.

S. No.	Properties	FR4	Paper	LS-EC substrates(this work)
1	**Material**	Glass + epoxy (*83*)	Cellulose (*84*)	Natural lignocellulose + etherified cellulose
2	**PCB fabrication**	Subtractive (*18*)	Additive (*38*)	Additive (Fig. 3)
3	**Carbon footprint**	17.7 kgCO_2_e/m^2^ (*85*)	~4.7 kgCO_2_e/m^2^ (*86*)	~1.64 kgCO_2_e/m^2^
4	**Amount of material for 25% conductive area on 1-m**^**2**^ **substrate**	0.117 kg (removed via pollutive chemical etching) (*83*)	0.057 kg (added via printing with minimal waste) (*83*)	Slightly lower than paper because the substrate does not absorb the ink into fibers
5	**Metal recovery**	Difficult (*87*)	Difficult (*47*, *83*)	Feasible (fig. S16)
6	**Suitability for reflow soldering**	High (*88*)	Low (*48*, *89*)	High (Fig. 3)
7	**Glass transition temperature**	135°–180°C (*90*)	~174°C (*91*)	~95°C
8	**Moisture resistance**	High	Low	High
9	**Dielectric constant**	4.6 (*92*)	2–4 (*93*)	3.4

Leaftronics offers three sustainability breakthroughs that might be decisive for industrial adoption of the technology. First, Leaftronics is compatible with additive metal deposition techniques such as inkjet or screen printing, which significantly cuts down on CO_2_ emissions during PCB fabrication (see the Supplementary Materials on ink testing and sustainability analysis). Second, Leaftronic substrates (like the one in this publication) can withstand temperature profiles above 200°C, which enables the use of reflow soldering and, thus, a smooth ingress into existing electronics mass production setups. Third, in contrast to paper, where the metal ink is absorbed by cellulose fibers, Leaftronic substrates do not absorb the ink deposited on them. Thus, precious metals (here, Ag) can be removed seamlessly and reused for the formulation of new inks (see fig. S16 and description) along with the removal of electronic components (Fig. 3A). These factors amplify Leaftronics’ sustainability benefits compared to traditional PCBs and alternative bio-sourced substrates such as paper. Moreover, compostability and reusability of metals open up a plethora of different upcycling and recycling strategies (elaborated in fig. S16 and associated description).

At the EoL, the most important aspects to consider for environmentally sustainable PCBs are biodegradation, upcycling of precious metals, and the recovery of electronic components. Any one of these, if not accounted for in a newly proposed substrate, can contribute significantly to CO_2_ emissions (*18*, *47*, *48*) and, for that reason, have been rigorously tackled within this work.

## DISCUSSION

This work reports a facile technique to implement biodegradable polymers with low thermal resistance for use in high-temperature applications without chemically modifying the polymers or affecting their biodegradable nature. What is shown is that quasi-fractal structures can create a coalescing scaffold for such materials when they lose their mechanical consistency above their *T*_g_ values or even above their melting points. We show that, instead of synthesizing such complex scaffold structures in the laboratory, one can instead implement ready-made, highly effective, quasi-fractal structures from nature. Natural leaves contain lignocellulose-based vasculature that, when extracted, can effectively function as quasi-fractal scaffolds that can coalesce polymer solutions to form films. Not only does this present an extremely effective method to achieve freestanding films of functional materials via simple dip coating, but the lignocellulose scaffolds being also biodegradable, flexible, and chemically stable ensure that polymers dissolved in myriad solvents could be implemented without changing the biodegradability or flexibility of the resulting substrate.

We perform extensive testing of a leaf-skeleton reinforced, solvent-dissolved biodegradable polymer, EC, to show the effectiveness of this technique. We also demonstrate a method to make such substrates smooth enough for thin-film device fabrication applications while showing that their thermal resilience is retained even during commercially equivalent reflow soldering of thick-film electronics. We demonstrate how the surface of such reinforced substrates can be functionalized for myriad application scenarios and deposition processes (such as PVD, inkjet printing, and screen printing).

When functionalized with CS, PVD experiments on LS-EC-CS substrates indicate that, although the CS topcoat might be perfectly suitable for the deposition of metals such as Au, it might be detrimental toward the deposition of oxidizable metals such as Al or Ag (fig. S3). In addition, because screen printing (being a contact deposition process) results in better ink adhesion as compared to inkjet printing and PVD (even with the CS adhesion promotion layer), we quantified the adhesion of screen-printed Ag on both the LS-EC and LS-EC-CS substrates using the IPC-TM-650 standard tape test (fig. S12). This resulted in the finding that, although the ink adhesion is excellent in both cases, mechanical failure could occur at the EC-CS interface where the entire CS layer intended for surface property tuning of the LS-EC substrate can come off along with the intact printed Ag layer (fig. S12B). This indicates that, although the surface of the LS-EC substrates can be functionalized with CS, the process requires further fine-tuning in terms of chemically improving the adhesion of CS to LS-EC substrates as has been shown previously (*26*, *27*, *49*). In terms of the measurement of the *T*_g_, the complexity associated with accurately measuring this value for semicrystalline polymers such as EC, especially when reinforced with a lignocellulose scaffold, is high. The question whether the *T*_g_ is changing in the presence of the quasi-fractal scaffold is pertinent, and differential scanning calorimetry (DSC) measurements (Table 1 and fig. S17) indicate that more attention needs to be paid to this aspect while considering substrate formation within the gamut of Leaftronics. In addition, although the materials used to fabricate our substrates are separately known to be biodegradable (*50*), a certified biodegradation test of the substrates was not undertaken here, and the biodegradation was only studied as a factor of degradation over time in a compost environment. Such biodegradation tests on these substrates, especially with thin-film organic devices fabricated with biodegradable, thermally resilient encapsulation, would open further vistas of environmentally sustainable electronics.

Considering the scale at which pollution from electronic waste is rising, research into low carbon footprint substrates that are biodegradable, exhibit moderate thermochemical stability, and maintain the flexibility and transparency of plastics is critical. This work paves a new path toward such a goal by showing that, although biodegradable polymers have low thermal tolerance, they can be reinforced using nature-derived quasi-fractals that instill thermal resilience onto films of such materials. Using EC as an example, we fabricate substrates that already show potential for replacing glass and plastic foils in organic electronic and hybrid electronic applications. The LS-EC substrates have compelling mechanical properties, placing them ideally between inorganic substrate medium like PDMS and organic medium like paper. The substrates achieve excellent light transmittance values between 80 and 85% and an RMS surface roughness below 6 nm combined with good ink adhesion properties and high flexibility. Microcontroller-based programmable hybrid circuitry using commercially used reflow soldering methodology along with fully functioning flexible organic devices like high-performance printed OECTs and PVD-deposited OPDs has been demonstrated here. The potential for substrate biodegradability is exemplified via composting, and the ease with which soldered electronic components and the metal particles used to fabricate circuit interconnects can be extracted and recycled from the substrate using biocompatible mild acids at room temperature is demonstrated. These results show that the entire gamut of biodegradable polymers that have been difficult to experiment with up until now due to their low-temperature tolerance may now be open for novel experimentation as functional films in commercial inorganic electronics and future flexible organic electronic applications. The aforementioned vast experimental possibilities, along with the associated CO_2_ reduction and further sustainability parameters, make Leaftronics an essential supplement toward the final goal of responsible electronics.

## MATERIALS AND METHODS

EC [48.0 to 49.5% (w/w) ethoxyl basis], CS (molecular weight, 310 to 375 kDa), 2-butoxyethanol (spectrophotometric grade, ≥99.0%), dimethyl sulfoxide (DMSO) (≥99.7% assay), Triton X-100 (polyethylene glycol *tert*-octylphenyl ether), phosphate-buffered saline (pH 7.2, sterile filtered), sodium carbonate decahydrate (Na_2_CO_3_·10H_2_O) (>99.0% trace metals basis), and polyethylenimine (PEI) (branched, average molecular weight of ~25,000, determined via light scattering) were procured from Sigma-Aldrich, Germany. Screen printable PEDOT:PSS ink (5 wt %, ≥50 Pa·s) and inkjet-printable Ag nanoparticle ink were procured from NovaCentrix (JS-A102A), and Ag micro flakes (10 μm) were also procured from Sigma-Aldrich, Germany. PEDOT:PSS dispersion for inkjet printing (Clevios PH1000) was procured from Heraeus Epurio, Germany. Enzymatic biodegradable detergent was procured commercially from the bio detergent supplier Ecover. Low-temperature solder-paste SMDLTLFP containing 42% tin (Sn), 57.6% bismuth (Bi), and 0.4% silver (Ag) was procured from Chip Quik. The compost used is formed from generic domestic bio matter.

### Solution preparation

CS solution that could be easily spin coated at 1000 rpm was prepared by dissolving 98 mg of CS in 10 ml of 100 mM acetic acid (CH_3_COOH) solution under constant stirring at 90°C overnight. EC is cellulose-derived solution processable polymer that is commercially synthesized by reacting partially depolymerized cellulose with ethyl chloride. The polymer solution was prepared by dissolving 8 g of EC in 36 ml of 2-butoxyethanol and leaving the mixture under low stirring at 90°C for 5 hours. This solution was plasticized by adding 1.76 g of edible rapeseed oil during stirring, and the temperature was raised to 170°C for 2 min to allow for proper mixing of the oil beyond the *T*_g_ of EC. The temperature was subsequently brought down slowly to 90°C with stirring allowed to continue for another 15 min for a thorough blend.

Silver ink for screen printing was prepared by dispersing 2.6 g of Ag (~10-μm flakes, Sigma-Aldrich) in 2 g of EC solution followed by stirring at room temperature for 5 to 10 min until a uniform dispersion of Ag was achieved. 2-Butoxyethanol (0.55 g) was added to the mixture and stirred manually. Last, 3% (by weight of the final solution) of PEI was subsequently added to the dispersion and manually stirred with a spatula before ultrasonicating the preparation for 20 min. Satisfactory ink consistency was achieved after homogenization at 1000 rpm for 1 min.

Commercially available PEDOT:PSS ink for inkjet printing, Clevios PH1000, was modified by diluting the ink in a 1:1 ratio with deionized (DI) water. DMSO [5% (v/v)] was added to this solution and stirred for 1 min before lastly adding 1% (v/v) of Triton X-100 while stirring.

### Thermomechanical testing

The coefficient of thermal expansion (CTE) was determined using a thermomechanical analyzer (TA Instruments TMA Q400) (fig. S9) in tensile mode on three material samples with two preloads (0.02 and 0.05 N) between −20° and 100°C. The modulus of elasticity and elongation at break were determined in the tensile test using a Zwick “UPM zwicki 2.5” tensile testing machine with mechanical clamping jaws and a 10-N load cell in accordance with the DIN EN 527-1/2 standard. The displacement was determined via crosshead travel. Because of the inhomogeneous thickness distribution and the morphology of the test specimens, it was only possible to determine the test specimen cross section roughly, and the results should be regarded as conservative because the thickness determination with the thickness gauges used could essentially only be determined on the thickest areas of the test specimens. An exact determination of the thickness would reduce the cross section and increase the modulus and tensile strength accordingly. However, it was possible to determine the width of the test specimens precisely. The *T*_g_ was measured using DSC and followed the ISO 11357 standard.

### Device characterization

Transmittance measurements are performed in an integrating sphere using a UV/visible/near-infrared spectrophotometer SolidSpec-3700 from Shimadzu, Japan. The EQE measurements are acquired in air using the light of a xenon lamp (Oriel Xe Arc-Lamp Apex Illuminator), optically chopped at 172 Hz and coupled into a monochromator (Newport Cornerstone 260 1/4 m monochromator, USA). The monochromatic light output of the monochromator is focused onto the device, and its generated photocurrent is measured under short-circuit conditions. The signal is amplified by a current preamplifier and then fed to a lock-in amplifier (Signal Recovery SR 7265, USA) with a time constant of 100 ms. The flux of incident photons is measured using a calibrated Si photodiode (Hamamatsu S1337, Japan, calibrated by Fraunhofer Institut für Solare Energiesysteme), and, subsequently, the EQE is calculated by dividing the photocurrent of the device by it. The current density–voltage characteristics are measured with a source-measure unit (Keithley 2400, Keithley Instruments, USA) under illumination and in darkness. The devices were illuminated with an intensity of 100 mW/cm^2^ (AM 1.5 G) provided by a sun simulator (Solar Light Company Inc., USA). The intensity is controlled by a Hamamatsu S1337 silicon photodiode. The dark current is derived at 0 V to determine the shunt resistance *R*_Shunt_ and, accordingly, the thermal noise current *I*_thermal_, where *k*_B_ is the Boltzmann constant, *T* = 296 K is the temperature, and Δ*f* = 1 Hz is the electrical bandwidth

$$I_{\text{thermal}}=\sqrt{\frac{4k_{B}T∆f}{R_{\text{Shunt}}}}$$

O_2_ plasma treatment for the pretreatment of borosilicate glass substrates was performed using Diener electronic ATTO plasma-cleaner procured from Diener electronic GmbH & Co. KG, Germany. Inkjet printing was performed using a Dimatix Materials Printer DMP-2850 from Fujifilm, Germany. The printer was used to deposit source, drain, and gate electrodes using nanoparticle Ag ink and for the deposition of PEDOT:PSS ink for the fabrication of OECTs. Inkjet printing of Ag was also used to fabricate PCB interconnects to form hybrid circuitry on the LS-EC-CS substrates.

Screen printing was performed using an Ekra E2 screen printer from ASYS Automatisierungssysteme GmbH, Germany. The technique was implemented to fabricate PCB interconnects for hybrid circuits on the LS-EC as well as LS-EC-CS substrates with a circuit interconnect layout identical to that used for the inkjet-printed hybrid circuits. A 90-40 screen was used to deposit the Ag ink, resulting in a final thickness of the Ag layer of about 65 ± 3 μm and a sheet resistance of about 1 ± 4 ohms/□ using the ink formulation developed in this work. 

The homogenizer used for the thorough mixing of Ag ink prepared in this work was a high-speed stainless steel VEVOR FSH 2A mechanical homogenizer. The homogenization was carried out for 1 min at 1000 rpm to ensure that friction against the Ag microparticles did not overheat the ink.

Physical vapor deposition (PVD) for the fabrication of OPDs was performed using thermal evaporation of metals and organics onto the LS-EC substrates under an operating pressure of 10^−7^ mbar with the layer thickness monitored using a quartz crystal microbalance. AFM was performed using the Flex-Axiom from Nanosurf AG, Switzerland, and the process was conducted in tapping mode. The long cantilever AFM tips with aluminum reflective coating (TAP-190Al-G) were purchased from BudgetSensors.

SEM was performed to record the cross-sectional image LS-EC substrates and LS-EC-CS substrates. For this, the substrates were cut with a pair of standard scissors because these were seen to cause less compression of layers as compared to using a scalpel. For SEM, a conductive surface is needed, and, therefore, 4 nm of carbon was deposited via pulsed carbon rod deposition in a vacuum chamber. The image was recorded using a Zeiss GeminiSEM 500, which was operated at a 3-kV acceleration voltage and under a 10^−5^-mbar pressure. Secondary electrons were detected with a detector placed on the side of the focusing column.

Profilometry was performed using the Dektak 150 Profilometer to measure the thickness of printed layers as well as the large-scale surface topology (micrometer to millimeter range) of the substrates reported in this work. The instrument has a maximum scan length range of 55 mm with 60,000 data points per scan.

### Experimental design

#### LS harvesting and coating

Magnolia, apart from having dense, rugged venation in its leaves, is also known for its hardiness (they can be grown in locations with seasonal snowfall), affordability, and availability in most continents as a native species or as cheap ornamental plants for purchase (samples for initial testing were procured from flowering trees in May from the Technische Universität Dresden campus). Apart from this, the decision to select Magnolia leaves here stems from the fact that relatively fleshy leaf species (such as Magnolia) tend to be easier to extract skeletons from and that Magnolias have been prominent in recent studies that have focused on gaining a deeper understanding of leaf-skeletons (*51*) and their potential applications (*52*, *53*).

Mature magnolia leaves (Fig. 1A.1) were washed under running water before being placed in an ultrasonic ethanol bath for 10 min to remove any impurities. Leaves were considered “mature” if they had achieved the maximum average size; were healthy; and were not old enough to have marks, damage, or discoloration. The selected leaves were cut to a size of 2.5 cm by 2.5 cm and subsequently boiled in water for 30 min. The samples were subsequently placed in a DI water aqueous solution of Na_2_CO_3_·10H_2_O (sodium carbonate decahydrate) and heated at a constant temperature of 90°C under stirring for 5 hours. The quantity of Na_2_CO_3_·10H_2_O to be used was determined by simply increasing the amount until no more of the compound dissolved. The heat, alkalinity, and fluid turbulence caused cellular damage and removal of the mesophyll, resulting in the solution turning dark. Hence, the leaves were filtered out from this solution and placed into a separate vessel with hot distilled water and subsequently placed into an ultrasonic bath for 20 min. The Na_2_CO_3_·10H_2_O treatment was repeated multiple times, and the needed repetitions can vary depending on not only the quantity of leaf cutouts being processed simultaneously but also the maturity of the leaves selected because older leaves lose their mesophyll more readily than younger ones, and this can be difficult to distinguish for the untrained eye while collecting. However, the process was repeated until the water no longer turned dark in color. The venation in the leaves was clearly visible at this point, and gentle brushing with gloved fingers was enough to remove excess biomass and expose the LS structure. The LS was subsequently submerged in commercially available biodegradable detergent solution and left overnight under stirring to remove any mesophyll structures adhering to the lignocellulose. The skeletons were subsequently washed in diluted (4%) hydrogen peroxide (H_2_O_2_) for 10 min, washed with DI water, dried, and lastly flattened with weights. The thickness of the final LS structures is about 150 μm on average as determined via micrometer measurements, and the size of the structures within is generally in the range of 150 μm.

The coating of the LSs (extracted using the aforementioned process) was performed manually using a dip-coating technique. The 2.5 cm–by–2.5 cm LS was manually slid into a petri dish containing EC solution (fig. S1A). Full submersion was ensured (while avoiding any bubble formation) by gently pressing the LS into the viscous liquid with a spatula. The LS was subsequently allowed to stay submerged for 30 s before being gently pulled out of the solution at a uniform velocity of about 1 mm/s using rounded tweezers. The now coated LS was passed over the edge of the petri dish while being pulled out to remove excess EC. The fully extracted EC-coated LS was held over the petri dish containing the EC solution for 10 s to further allow any excess EC to drip off before being placed on an uncoated glass substrate or a glass substrate coated with a thin layer of CS as explained in the next sections.

#### LS-EC-CS exfoliation

A thin layer of CS was spin coated (1000 rpm, 60 s) onto a borosilicate glass slide having the same dimensions as the LS (Fig. 1C) and dried at 90°C for 10 min. The freshly dip-coated LS (see the previous section) was placed on to this CS-coated glass (Fig. 1C), and the stack was heated at 90°C for 3 to 4 hours before being extracted and allowed to cool at room temperature. The naturally positively charged CS polymer layer binds favorably to the negatively charged EC layer during this heating process and the entire stack, i.e., the EC-coated LS along with the CS was, hence, manually peeled off from the glass surface using the edge of a spatula or blade (Fig. 1C.3).

#### LS-EC exfoliation

Smooth LS-EC substrates, i.e., without the CS layer, were prepared by first dip coating the LS structure as elucidated in LS harvesting and coating and then placing the same onto bare, uncoated glass slides. The glass slide carrying the LS-EC was subsequently dried at 90°C for 3 to 4 hours. At the end of this period, the hot dry samples were immediately quenched via placing them in a freezer (−30°C) for 1 min. The different rates of thermal contraction of the glass and the LS-EC hybrid material result in the weakening of the adhesive bonds. The substrates could manually be peeled off from the glass slides at this point using the edge of a spatula or a blade. Peeling off without the thermal quenching process is also possible using a sharp blade edge but risks damaging the substrate if not performed carefully. The surface has the same smoothness as the LS-EC-CS substrates (<3.3-nm RMS) when compared via AFM and is suitable for the fabrication of functioning OPDs (~257-nm theoretical device thickness). If needed, a CS topcoat can still be added to the LS-EC substrate via spin coating.

### Bending and temperature measurements

**Fig. 5.**
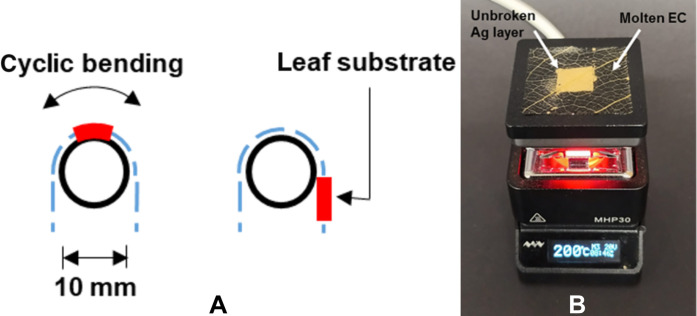
Setup for bending and temperature stability measurements. (**A**) An illustration of the bending that the substrates were exposed to (8000 cycles). (**B**) An LS-EC substrate with a 1 cm–by–1 cm screen-printed layer at 200°C.

A 2 cm–by–2 cm area of Ag was deposited onto two 2.5 cm–by–2.5 cm LS-EC substrates using a 165-31 screen and a 61-64 screen. A resulting layer thickness of ~9 μm (measured using a profilometer) could be achieved upon printing with the 165-31 screen, and a thickness of ~35 μm could be achieved by printing the same design with a 61-64 screen. The printed samples were dried at 90°C for 20 min and left at ambient room temperature overnight before bending experiments were performed the following day. The bending process implemented is shown in Fig. 5A and was achieved using a self-built cyclic bending apparatus implementing a 1-cm-diameter cylindrical roller. The results of the bending experiments are shown in Fig. 2C.

A 1 cm–by–1 cm^2^ area of Ag was screen printed onto three separate LS-EC substrates using a 90-40 screen (~25-μm layer thickness) before placing them onto a hotplate with the corresponding sheet resistance measured at defined temperature intervals. The results of the temperature tests are shown in Fig. 2F, and the substrate could successfully keep the molten polymer coalesced at 200°C, resulting in an unbroken Ag layer. Further temperature experiments are details in figs. S7 and S8.

### Circuit soldering

Circuit tracks were fabricated on the LS-EC-CS substrate using additive techniques, and both inkjet printing (Fig. 3A) and screen printing (fig. S16A) were tested for the deposition of the Ag interconnects. The Ag-printed LS-EC-CS was temporarily glued onto a glass slide for mechanical support using a 50-μm-thick double-sided tape. A non-lead solder paste was chosen for the process (42% Sn, 57.6% Bi, and 0.4% Ag) and was deposited onto the appropriate locations where components were to be affixed on the circuit tracks. An SMD ATTINY85 MCU in an SOIC8 package was chosen for the experiments with two digital output pins programmed to sequentially output increasing and decreasing PWM signals. These signals were fed into two commercial SMD LEDs, respectively, with 100 ohms (1206 package) current limiting series resistors. The components were placed by hand and the hybrid PCB was fed into a commercial reflow oven (T962A reflow station) with a heating profile selected such that the temperature did not exceed 250°C for more than a minute. The solder was seen to easily reflow and create a permanent joint between the components and the printed Ag layer without causing any noticeable damage to the substrate or creating any shorts. The circuit was subsequently powered with a 3-V dc supply for testing.

### Device fabrication

#### Organic electrochemical transistors

The OECTs were fabricated using inkjet-printing on LS-EC-CS substrates. The LS-EC-CS substrates were cleaned in an ultrasonic bath with DI water for 1 min before being dried under a nitrogen (N_2_) gas flow. The samples were subsequently glued on to 2.5 cm–by–2.5 cm glass slides for mechanical support before inkjet-printing was undertaken. In terms of dimensions, the channel length was maintained at 200 μm with the channel width being ~500 μm. The gate-channel distance was ~250 μm. The electrodes were formed by inkjet-printing water-based Ag nanoparticle ink, and the channel was formed from inkjet-printing PEDOT:PSS (Clevios PH1000) ink.

#### Organic photodiode

The OPDs were fabricated by a thermal evaporation vacuum system (Kurt J. Lesker, UK) with a base pressure of less than 10^−7^ mbar. Before deposition, glass substrates were cleaned for 15 min in different ultrasonic baths with *N*-methyl-2-pyrrolidone solvent, DI water, and ethanol, followed by O_2_ plasma for 10 min. The LS-EC substrates were cleaned in an ultrasonic bath with DI water for 1 min before being dried under an N_2_ flow. The samples were subsequently glued on to 2.5 cm–by–2.5 cm glass slides using 50-μm-thick double-sided tape for mechanical support and heated overnight at 60°C in an oven before being placed under ~10^−6^-mBar pressure for 24 hours for outgassing before being transferred to the thermal evaporation vacuum system for PVD. This ensures that any moisture absorbed into the surface during storage or preexisting solvent residue is removed from the substrates before device fabrication. The device stacks of the OPDs are documented in fig. S15. The 0.78-mm^2^ effective active area was defined by the intersection of the bottom and top contact.

### Statistical analysis

All the relevant SD values for the thermomechanical measurements of the substrates are added in Table 1. The SD for the elastic modulus and the elongation was determined on eight separate samples, and the thermal expansion was measured on five samples before the SD was calculated. The measurement of the bending stability of the substrate (Fig. 2E) was performed on two samples with screen-printed Ag layers of different thicknesses (9 and 35 μm). The measurement of the change in sheet resistance of a screen-printed layer on the LS-EC substrate with respect to temperature was performed on three different samples from three separate batches and the resulting SD is included in Fig. 2F. The CTE was measured on three different samples using a thermomechanical analyzer and the calculated SD of the measurements is shown in Table 1.

## Supplementary Materials

### The PDF file includes:

Figs. S1 to S18 Tables S1 to S3 Legends for movies S1 and S2 References
