# Supplementary material for: Leaftronics: Natural lignocellulose scaffolds for sustainable electronics
Source: Sci Adv. 2024 Nov 8;10(45):eadq3276. doi: 10.1126/sciadv.adq3276 (PMC11546746; doi:10.1126/sciadv.adq3276)
Supplement: Supplementary file 1 — Figs. S1 to S18 Tables S1 to S3 Legends for movies S1 and S2 References [file sciadv.adq3276_sm.pdf]

Science Advances
AAAS

Supplementary Materials for

Leaftronics: Natural lignocellulose scaffolds for sustainable electronics

Rakesh R. Nair *et al.*

Corresponding author: Karl Leo, karl.leo@tu-dresden.de; Rakesh R. Nair, rakesh_rajendran.nair@tu-dresden.de

Sci. Adv. **10**, eadq3276 (2024)
DOI: 10.1126/sciadv.adq3276

The PDF file includes:

Figs. S1 to S18
Tables S1 to S3
Legends for movies S1 and S2
References

Other Supplementary Material for this manuscript includes the following:

Movies S1 and S2

Dip-coating:

Although the substrates were dip-coated manually, the following description shows how the process can be optimized further to achieve a higher level of uniformity between different dip-coated batches.

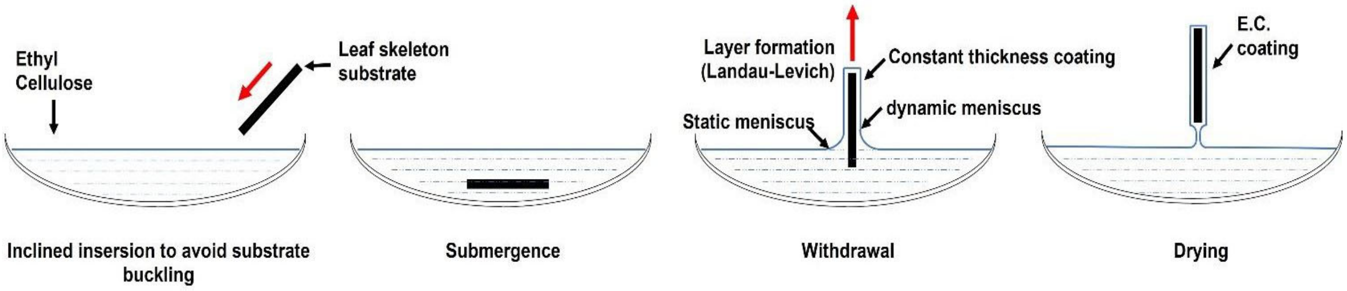

Figure S1: Dip-coating procedure

Here, the dip coating process was initiated by manually sliding in the 2.5 x 2.5 cm LS into a petri-dish containing highly viscous ethyl cellulose solution. The substrates were also gently pressed with a spatula to achieve complete, uniform submergence. Once the LS was fully submerged, it was pulled out slowly in order to achieve a uniform coating proximal with the Landau-Levich regime [94]. The Landau-Levich regime assumes a static-meniscus close to the surface of the liquid bath (Fig. 3A, main text), just above the static meniscus is the dynamic meniscus in which the liquid is sheared upwards, resulting in a uniformly thick coating on the substrate. This thickness (h) can be approximated [94] under low pulling velocities (~ 1 mm/s) as:

$$h \sim \sqrt[2/3]{\left(\eta V/\gamma\right)}$$

where h is the coating thickness, η is the fluid viscosity, V is the constant velocity of pulling and γ is the surface tension. However, since the Landau-Levich equation applies well to Newtonian fluids and the ethyl cellulose ink behaves more in the non-Newtonian shear-thinning regime, multiple approximations [94] may be selected from in literature due to the complex and non-linear rheological properties of non-Newtonian fluids. One extension to the Landau-Levich analysis by Gutfinger and Tallmadge [95] accounts for the shear-thinning of non-Newtonian fluids, where the coating thickness is given by:

$$h \sim \sqrt[\frac{1}{(2n+1)}]{\frac{k^2 V^{2n}}{\gamma^{1/2} \rho g^{3/2}}}$$

where k is the viscosity of the non-Newtonian fluid, ρ is the fluid density, and g is the acceleration due to gravity. For a Newtonian fluid, the viscosity wouldn't be a complex variant, therefore for n = 1 and k = η equation 2 reverts to the Landau-Levich regime. Figure 4 (main text) shows the L.S. substrate before and after the dip-coating with ethyl cellulose and subsequent curing under a vertical hot-air drying chamber.

Chitosan:

Gold (Au) PVD on glass generally results in a badly adhering layer that can be easily wiped off or scratched. Generally, a seed layer of chromium is used to improve the adhesion of gold on glass. The LS-EC-CS allowed for excellent adhesion of gold without the need for a chromium seed-layer (Fig. S2.A).

Furthermore, the adhesion enhancement of chitosan (CS) for metal layers was proven by the observation that Ag inks (water based nanoparticle dispersions) adhered much better with the CS coating on EC when compared to just EC. The adhesion improvement from functionalizing the surface with CS is shown in Fig. S2.B-C.

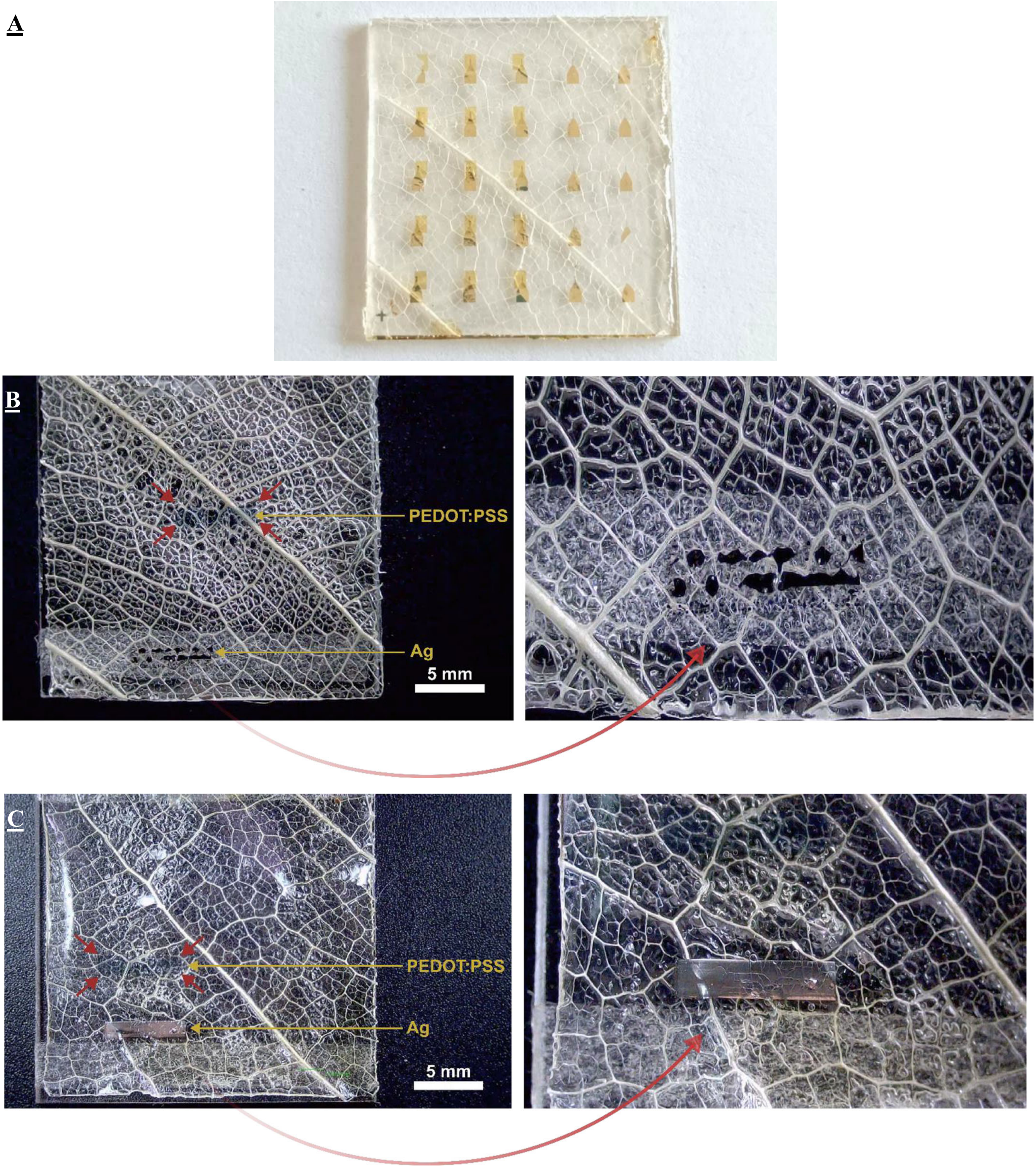

Figure S2: Material adhesion during PVD and inkjet printing on functionalized and non-functionalized substrates. (A) Chitosan functionalised substrate showing improved adhesion of gold during PVD without a chromium seed layer **(B)** Inkjet printing of water based Ag nanoparticle ink on LS-EC substrates showing impractically bad adhesion of ink. **(C)** When the LS-EC substrate

is functionalized with CS (i.e. the LS-EC-CS substrate) the adhesion improves remarkably as can be seen with the same Ag nanoparticle ink. (These tests were replicated numerous times with the same result)

It was noted that the PVD deposition of metals such as Aluminium and Silver inevitably led to oxidation in the presence of CS. As an example, **Figure S3.A** shows the PVD deposited Al layer coming out transparent with minimal conductivity immediately after PVD. The effect goes away when the chitosan layer is removed and PVD is performed only on LS-EC (**Figure S3.B)**. Further research is required to understand and explain this phenomenon fully.

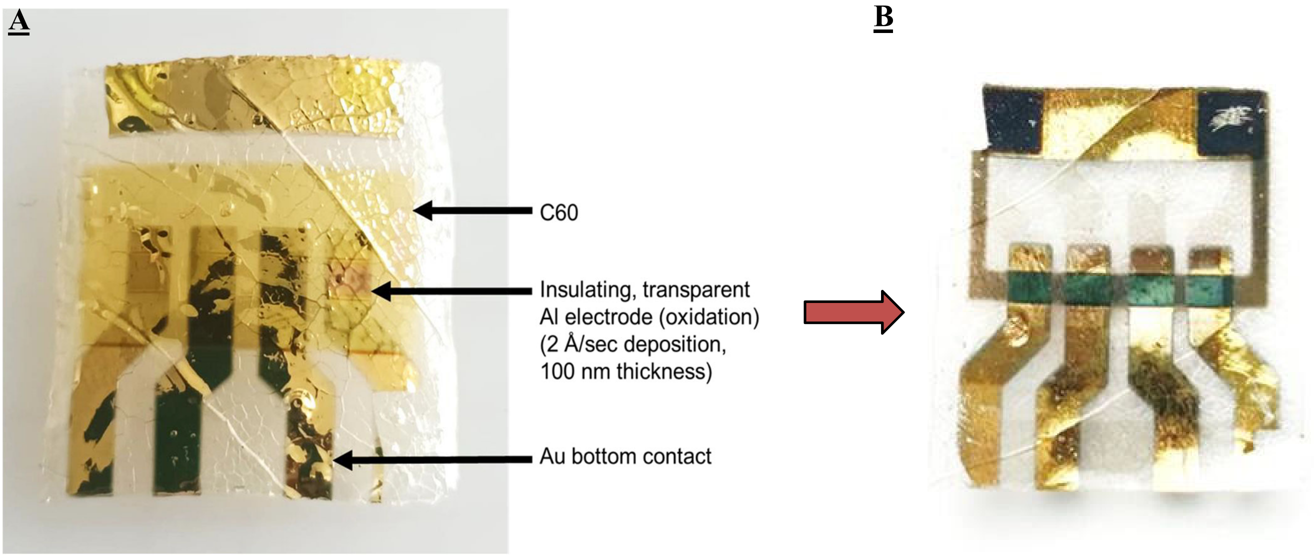

Figure S3: Problems associated with the functionalized substrate. (A) Anomalous oxidation of Al during PVD with chitosan top layer. The oxidation effect disappears without chitosan, as shown in (B)

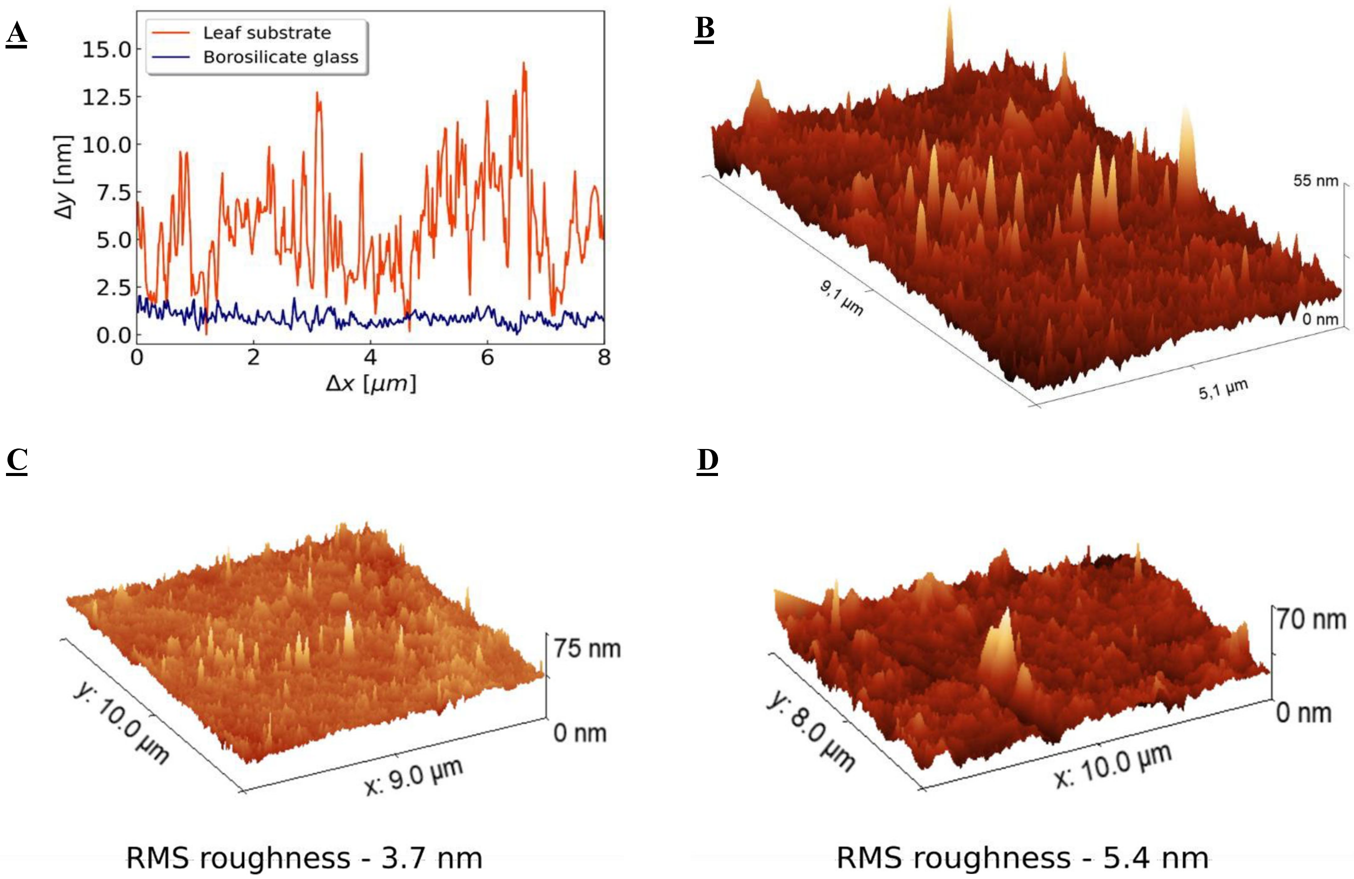

Figure S4: AFM measurements. (A) AFM measurement showing surface peak height in nanometers of an LS-EC substrate (red) and borosilicate glass substrate (blue) (B) Full AFM image of an LS-EC substrate (scanned area – 5 x 9 µm²) with RMS roughness of 3.3 nm. (C) Measurement of the same LS-EC substrate over a larger area (10 x 9 µm²) showing an RMS surface roughness of 3.7 nm (D) Roughness measurement performed specifically on top of a vascular structure on the LS-EC substrate showing an RMS surface roughness of 5.4 nm.

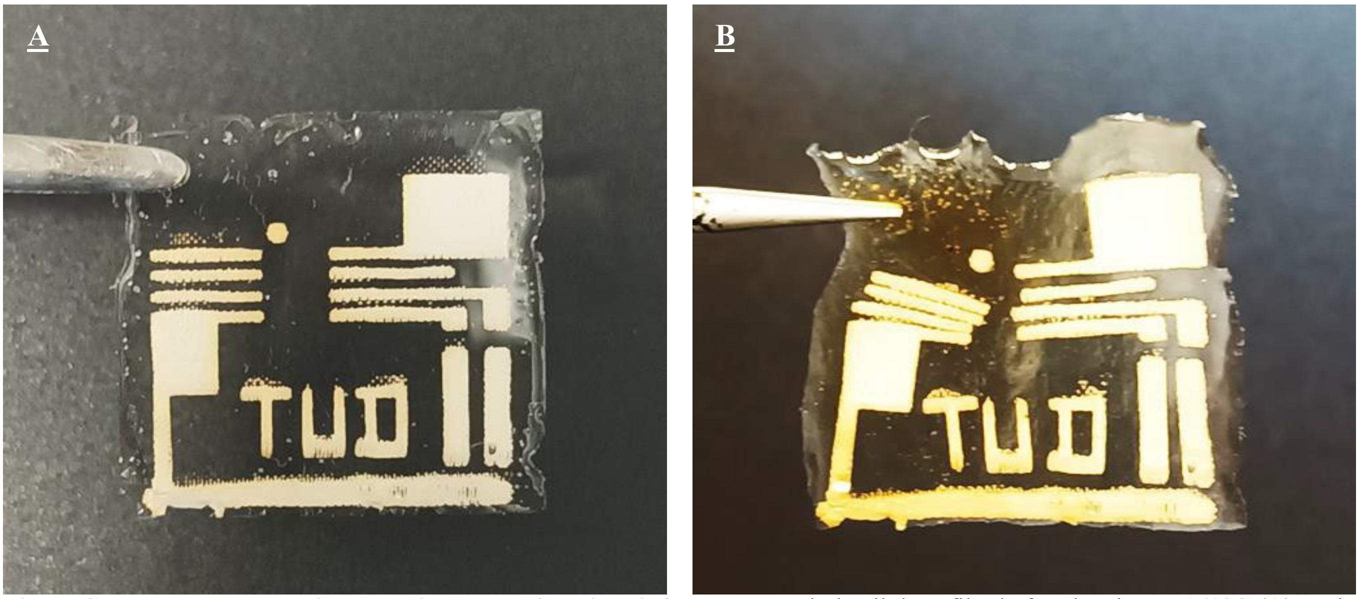

Figure S5: Temperature resistance without Leaftronic reinforcement. Ethyl cellulose film before heating to 160°C (A), and after heating to 160°C and subsequent cooling down to room temperature (B).

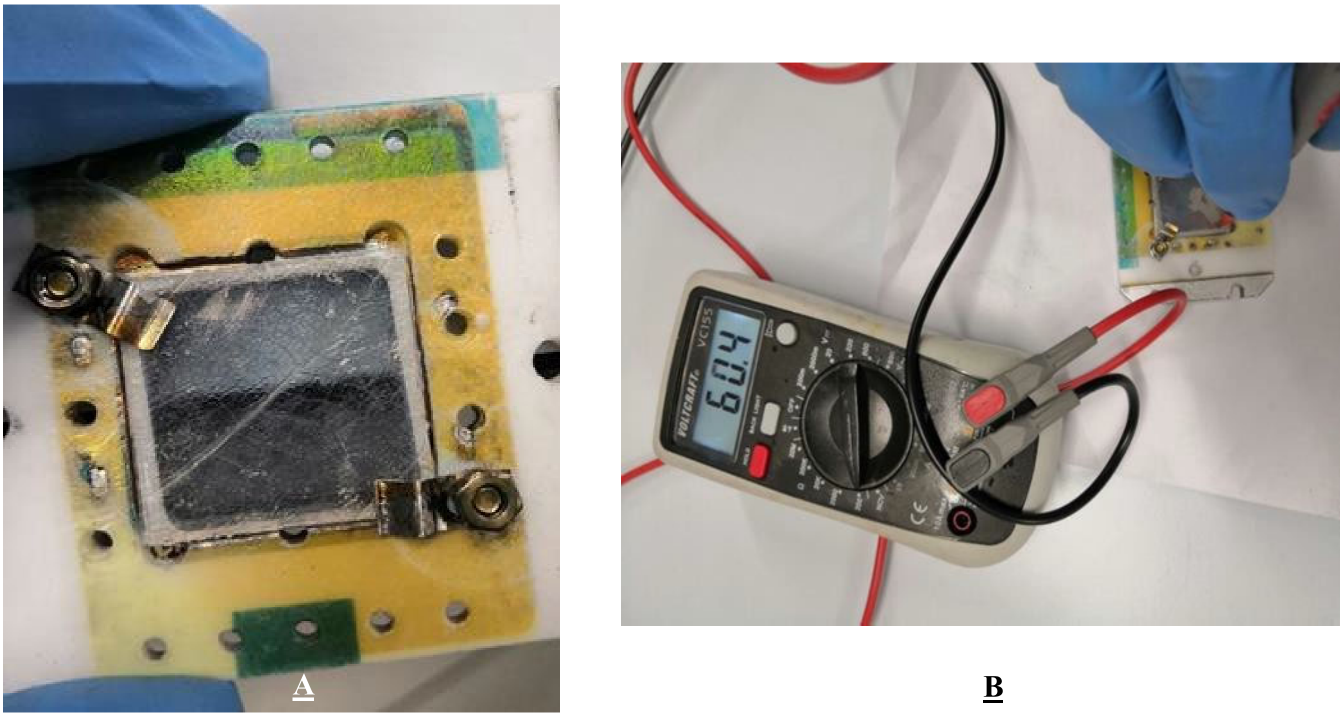

C

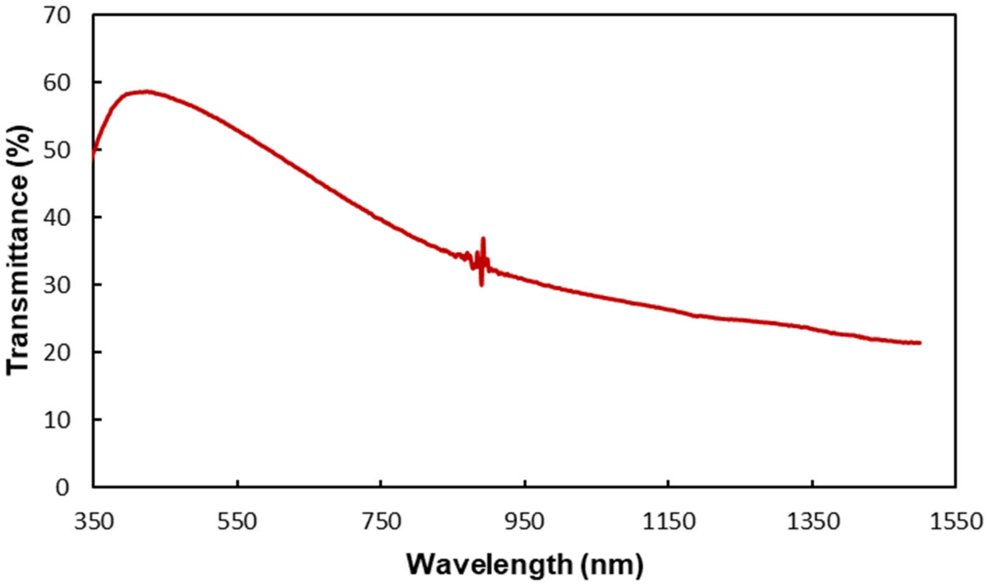

Figure S6: PVD based fabrication of TCEs. (A) Transparent conducting electrode fabricated using Cr (1.5nm)/Au (1.5nm)/Ag (10nm) on LS-EC substrate **(B)** Resistance measurement of the fabricated transparent electrode **(C)** Transmittance measurement of the same transparent electrode

Figure of merit (FOM) calculations for transparent conducting electrodes (TCEs) when performed for Cr (1.5nm)/Au (1.5nm)/Ag (10nm) electrodes fabricated via PVD on the leaf substrates give an FOM value of 0.49 Ω^{-1} based on the FOM Haacke High Resolution Figure of merit reported by Cisneros-Contreras et al.

The 'FOM Haacke High Resolution' ($FOM_{H\text{-}HR}$) is given as:

$$\phi_{H-HR} = \frac{T}{\sqrt[n]{R_{\square}}}$$

where 'ϕ_{H-HR}' is the FOM in units of Ω^{-1} and 'T' is the transmittance at 550 nm. '$R_{\square}$' is the measured sheet resistance with 'n' having a recommended value of 10. Considering the literature on transparent electrodes, FOMs greater than 0.45 Ω^{-1} are already deemed suitable for applications in photovoltaics, light emitting diodes (LEDs), gas sensors, thermal collectors etc. [96].

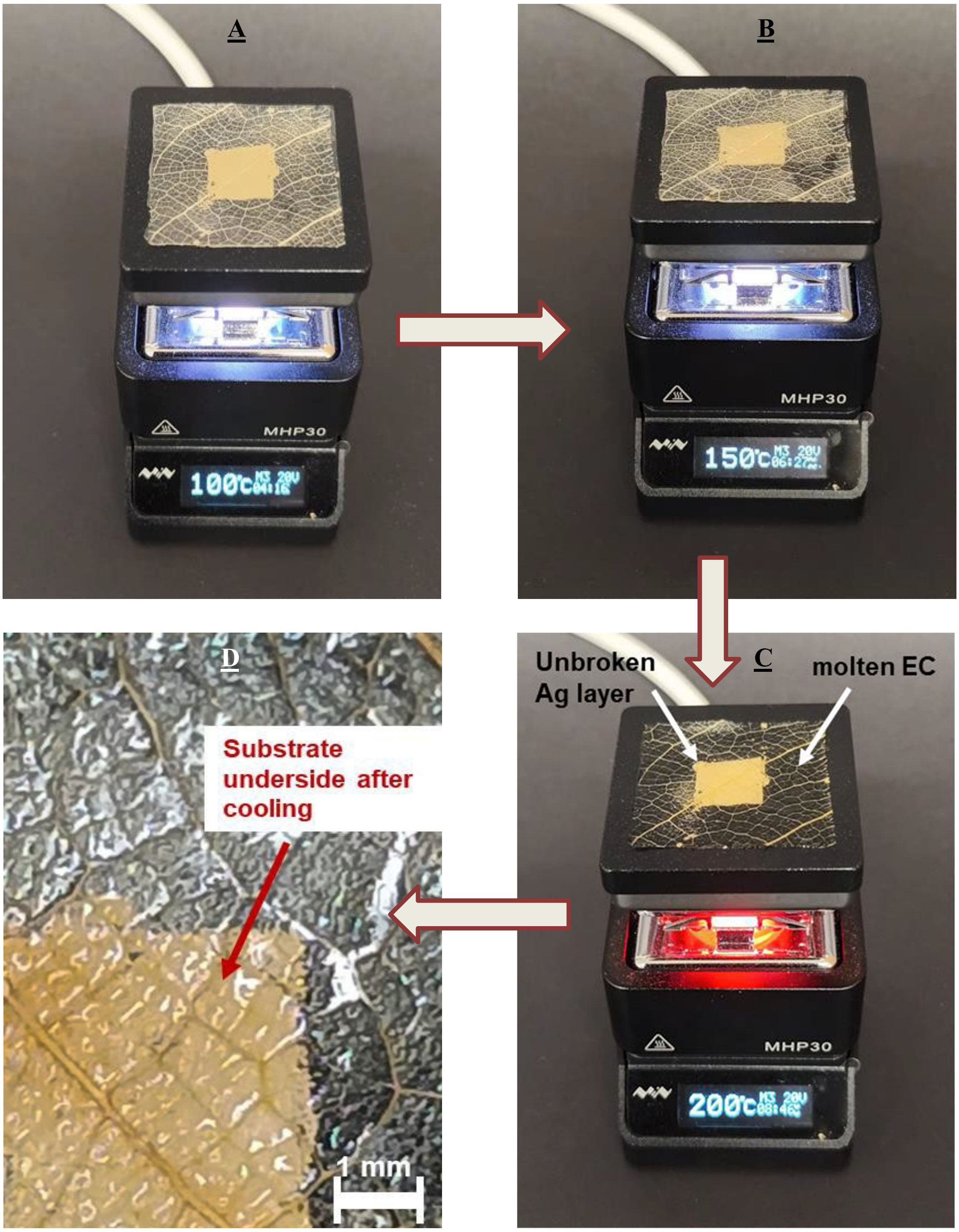

Figure S7: Printed layer stability on the Leaftronic substrates under elevated temperatures. Screen printed Ag on LS-EC-CS during thermal testing beyond EC's glass transition temperature (A) at 100°C. (B) at 150°C. (C) at 200°C with the Ag layer remaining undamaged even with a visibly melted EC layer (D) image of the underside of the substrate after cooling, showing the coalesced EC and unblemished Ag.

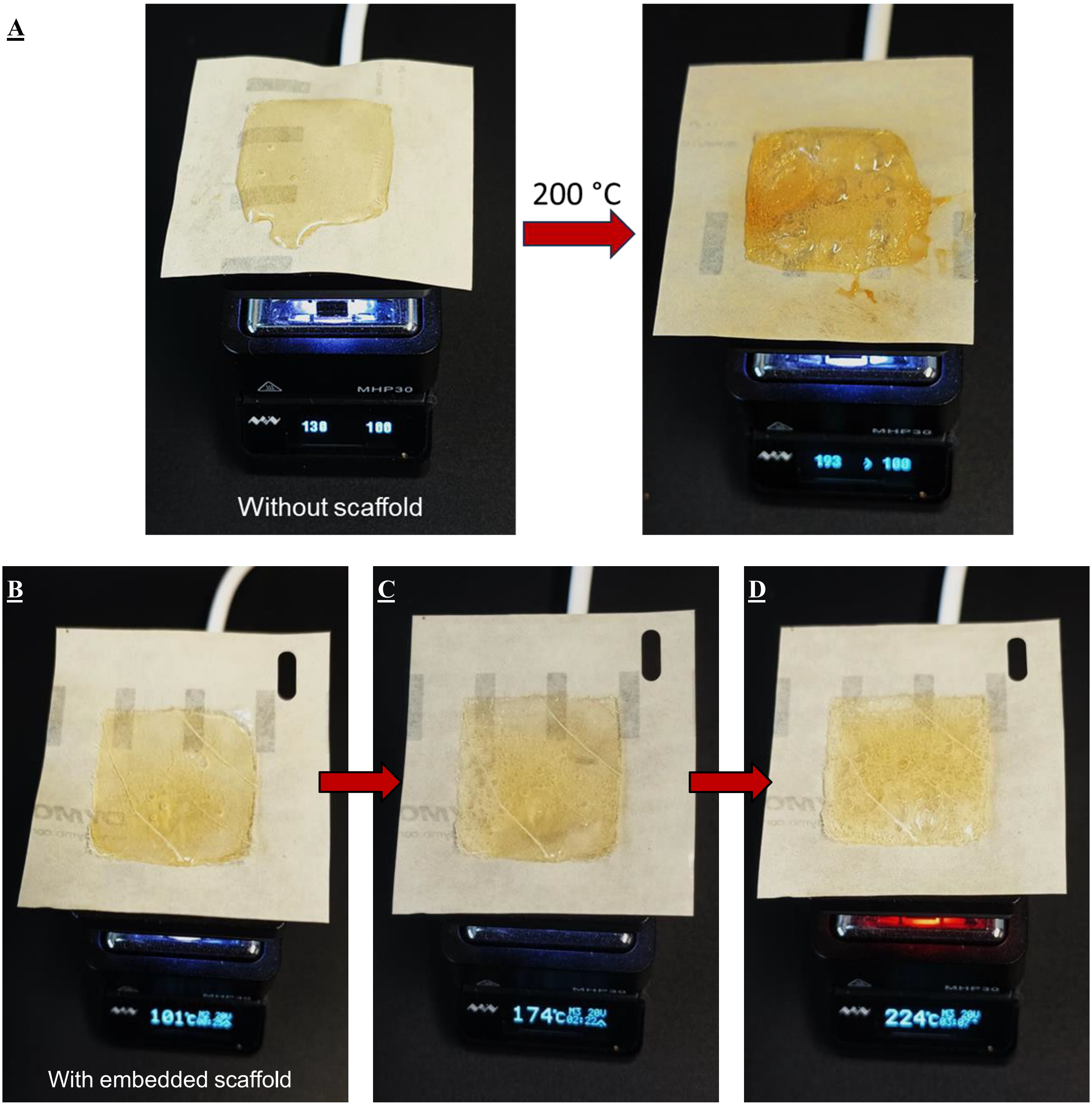

Figure S8: Leaftronic reinforcement of solution processed gelatine films. Gelatin films heated from room temperature to 230 °C showing (A) the film without a LS reinforcement melting and flowing across the non-stick paper at 138 °C and continuing its deterioration at 198 °C. (B) Films with an embedded lignocellulose scaffold remaining undeformed at 101 °C, at 174 °C (C) and 224 °C (D).

The lignocellulose scaffold provides thermomechanical stability to the film, however, the chemical properties such as the degradation temperature cannot be changed without chemical modification of the polymer itself.

Hence, gelatine, having a degradation onset temperature of 220°C, begins to form bubbles as can be seen in **Figure S8.D** even though the film structure remains stable unlike in **Figure S5.A**.

Table S1: LS-EC substrate properties compared with common heat-resistant, transparent substrates.

	Cost	Biodegradability	Transparency	Toxicity	Solderability
**Polyimide (PI)**	High	Non-biodegradable	< 60%	Non-cytotoxic [97]	< 300°C
**Polyethylene terephthalate (PET)**	Moderate	Non-biodegradable	~ 90%	Cytotoxic [98]	< 150°C [99]
**Functionalized leaf substrate (this work)**	Low	Biodegradable	> 80%	Non-cytotoxic [100]	< 250°C

Thermomechanical testing:

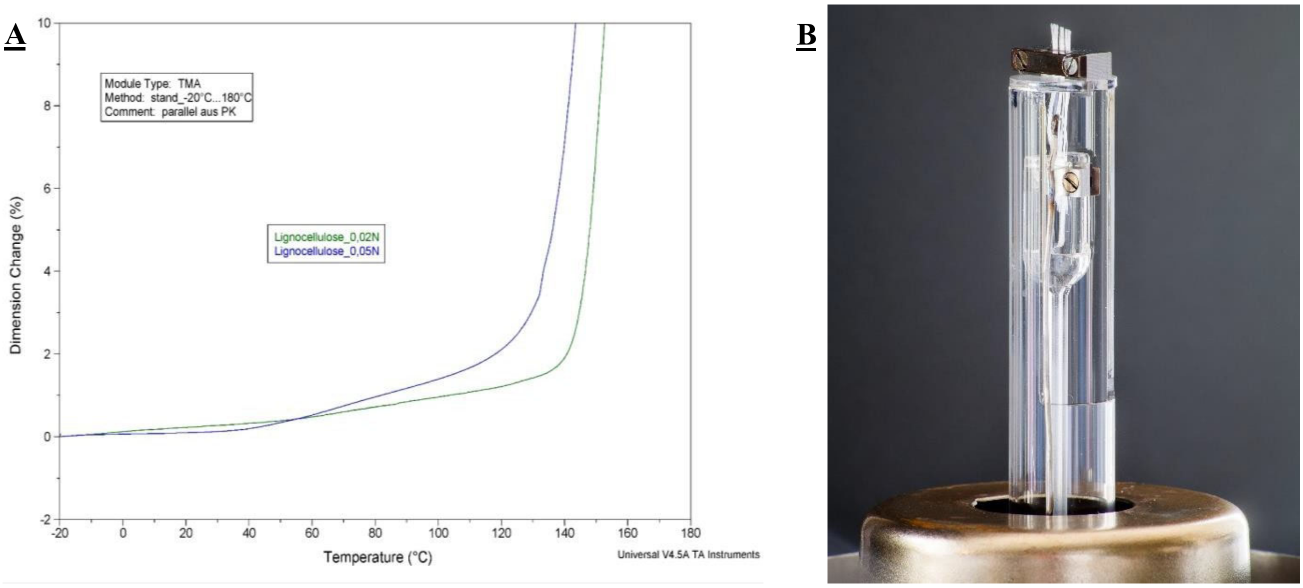

Figure S9: Thermal and mechanical tests of LS-EC substrates (A) Coefficient of thermal expansion, measured in-plane, using tensile loading of the substrates with two different masses (B) thermomechanical analyser - TA Instruments TMA Q400

Table S2: Stress, Strain and Elastic modulus measurements.

	Sample	ao	bo	Elastic modulus	sM	eM	sB	eB
		mm	mm	MPa	MPa	%	MPa	%
**Probe 1**	Pr.1_across	0.24	2	77.16537145	3.40697368	3.590765099	3.40697368	3.590765099

**Probe 2**	Pr.2_across	0.24	2	11.59390159	4.147567352	5.25632302	4.147567352	5.25632302
**Probe 3**	Pr.3_across	0.24	2	45.21445719	4.528689384	4.507512351	4.528689384	4.507512351
**Probe 4**	Pr.4_longitudinal	0.27	2	56.10990954	4.961096799	7.192516824	4.832415228	9.218251705
**Probe 5**	Pr.5_longitudinal	0.27	2	25.21713012	3.641444003	6.534270446	1.889931273	7.757436732
**Probe 6**	Pr.6_longitudinal	0.27	2	108.5647033	8.400270674	7.365940015	8.400270674	7.365940015
**Probe 7**	Pr.7_longitudinal	0.27	2	38.82393877	4.105982957	7.172230879	3.95150582	8.060547709
**Probe 8**	Pr.8_longitudinal	0.27	2	69.49888823	4.552797476	8.276037872	4.428990682	8.835310737

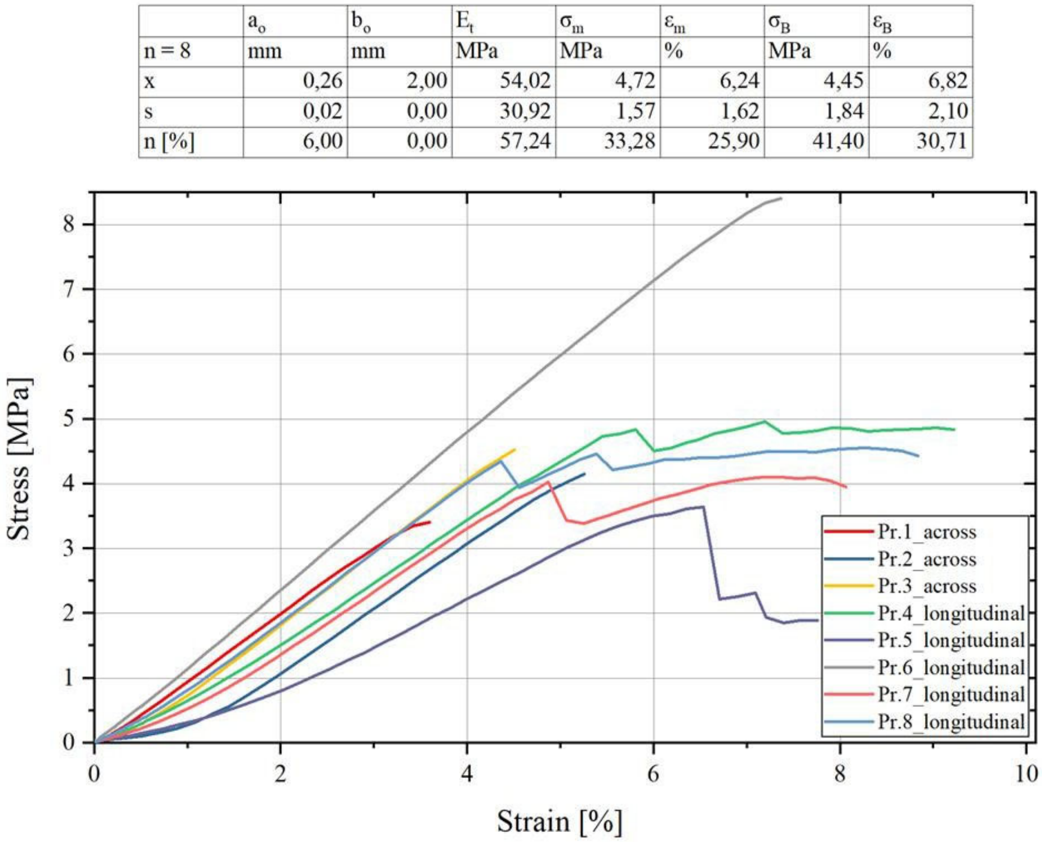

	a_o	b_o	E_t	σ_m	ε_m	σ_B	ε_B
n = 8	mm	mm	MPa	MPa	%	MPa	%
x	0,26	2,00	54,02	4,72	6,24	4,45	6,82
s	0,02	0,00	30,92	1,57	1,62	1,84	2,10
n [%]	6,00	0,00	57,24	33,28	25,90	41,40	30,71

Figure S10: Stress v/s strain curves for different leaf substrate samples

Ink testing:

Most functional inks contain organic solvents among other additives and the concern of potential substrate degradation during printing is tested by diluting commercial screen-printing Ag ink (see Section 4) with organic solvents such as 2-Butoxyethanol ($C_6H_{14}O_2$), acetone (CH_3COCH_3), isopropanol ($(CH_3)_2CHOH$) and ethanol

(C_2H_5OH). We observe that the CS top layer on the LS-EC-CS substrates keeps the material protected from most solvent related influence, which is probably a result of CS's naturally deficient interaction with organic solvents. However, since EC dissolves in many organic solvents, the influence of these solvent-modified inks on the substrate without a CS topcoat is also tested. For this test, screen-printing was performed to achieve an effective layer thickness of more than 30 µm (61-64 screen mesh) of organic solvent containing ink.

Surprisingly, apart from the lack of adhesion of water-based inks, the adhesion of solvent-based Ag inks remains unaltered and at times even improves with no noticeable damage to the LS-EC substrate. It is possible that the organic solvents contained in the ink partially disseminate into the surface of EC soon after the ink is freshly deposited. This subsequently allows Ag particles to embed and affix more extensively into the substrate surface, thus improving adhesion without any need for special treatment (**Figure S11.A**). Water-based Ag and Poly(3,4-ethylenedioxythiophene)-poly(styrenesulfonate) (PEDOT:PSS) inks adhere poorly due to the hydrophobicity of EC and the issue is made redundant with the CS topcoat which encourages chelation of metal ions and interacts well with acidic aqueous inks like PEDOT:PSS. For applications where the CS topcoat is not needed, plasma treatment of the LS-EC substrate also greatly enhances the adhesion of water-based inks.

In order to avoid any ecologically harmful chemicals, and since commercial ink suppliers do not disclose the chemical nature of their inks (solvents, co-solvents, thinners, binders, surface tension modifiers etc.), we also tested the substrate with in-house test formulations (details in Section 4.1). The results remained unchanged and hence confirm that the degradation resistance of the substrates is not a consequence of unidentified compounds in commercial inks. SEM images of the screen-printing performed on the LS-EC and LS-EC-CS substrates is shown in **Figure S11.B** and **Figure S11.C** where the exfoliated CS top coat is clearly visible on the LS-EC-CS substrates. Although the printing on both substrates was visibly good, the quality of adhesion was tested next and is elaborated upon in

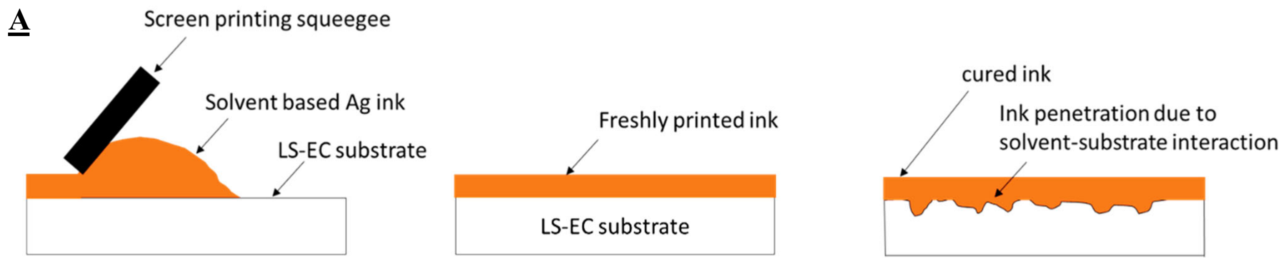

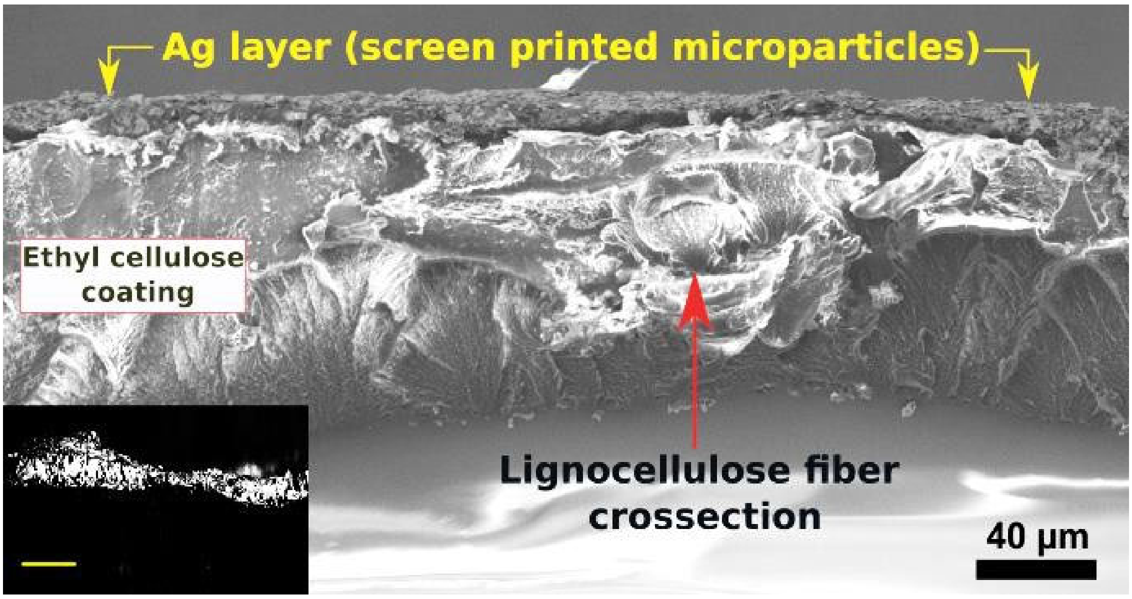

C

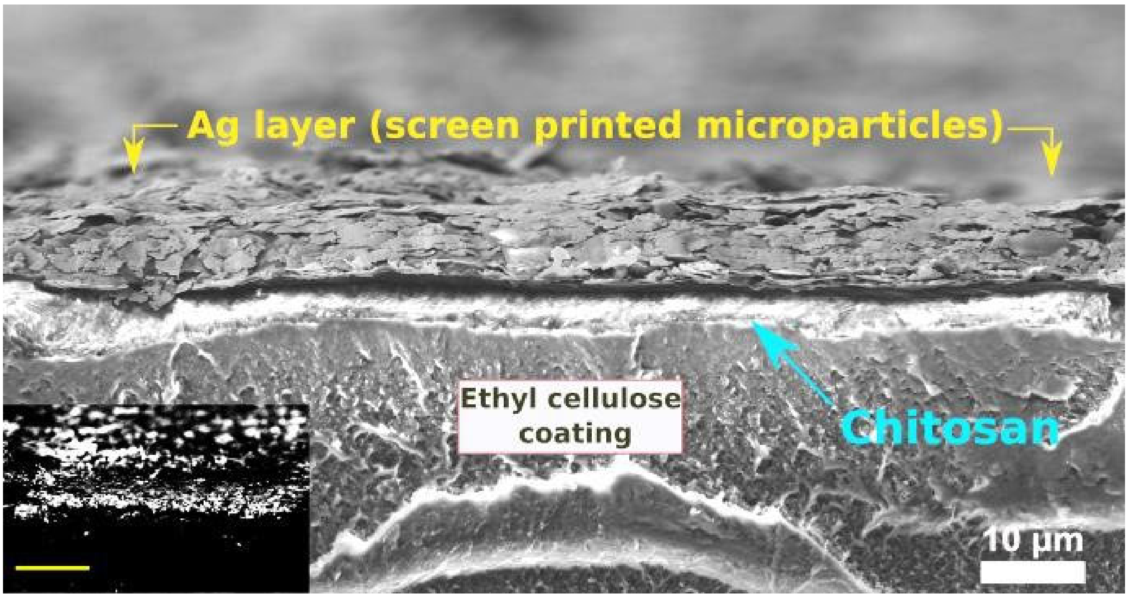

Figure S11: Screen printing on LS-EC and LS-EC-CS substrates. Improved adhesion of solvent-based ink on LS-EC substrates showing **(A)** Possible mechanism of adhesion wherein the ink solvents permeate the top layer of EC and increase the area of contact of the microparticles, hence improving adhesion **(B)** SEM image of screen-printed Ag microparticles on LS-EC substrate (**inset:** EDS imaging showing the Ag particles as heavier atoms, scale bar – 50 μm) **(C)** SEM image of screen printed Ag microparticles on LS-EC-CS substrate clearly showing the exfoliated CS layer (**inset:** EDS imaging showing the Ag particles as heavier atoms, scale bar – 50 μm).

Ink adhesion tests

A

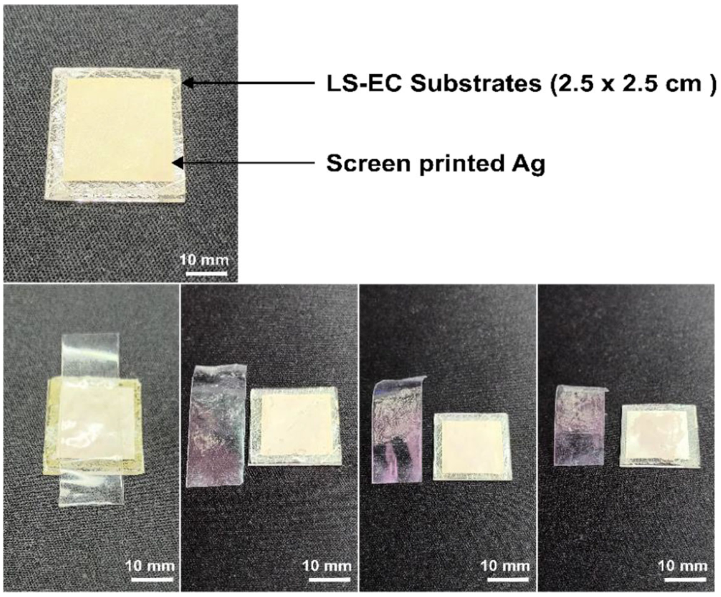

B

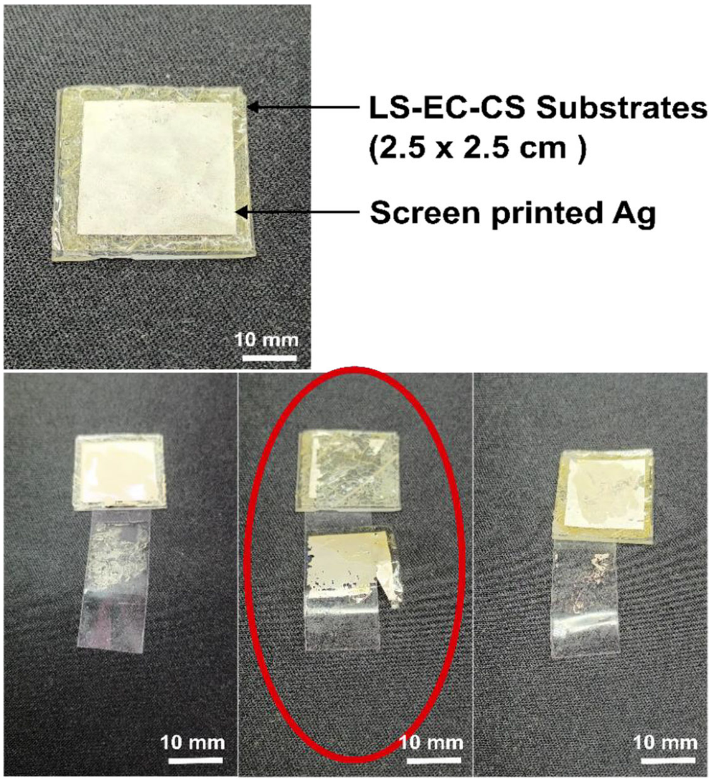

Figure S12: Ink adhesion tests. Testing based on IPC-TM-650 standard for 'Adhesion, Tape Testing' on **(A)** 2 x 2 cm^2 screen printed layer on the LS-EC substrates showing a printed sample (top), tape affixed on to the sample (bottom left) and the three

separate samples tested for the quality of ink adhesion along with the ripped-off tape placed next to them. **(B)** LS-EC-CS substrates with 2 x 2 cm^2 screen printed Ag layer along with the ripped off tape shown with the samples.

The quality of the printing was judged based on how well the screen-printed ink adhered to the substrate. For this reason, the IPC-TM-650 standard for the quantification of the quality of adhesion was implemented. The process involves the use of pressure sensitive tape (we used the recommended 3M 600 transparent tape) which is firmly pressed into the surface of a printed layer to avoid any air bubbles, after which it is pulled away rapidly at an approximately 90° angle to the surface. The time between application and removal of the tape is not allowed to exceed 60 seconds and a fresh strip is used for each test. At least three tests are required to be performed. After the removal, the tape is analysed for any residue of the ink material on its surface and if the printed layer is removed after this process, it is a mark of adhesion failure. Any slivers that get removed due to plating overhang is not considered a failure.

Figure S12.A shows the results of the adhesion tests performed on three LS-EC substrates and apart from minor residue (slivers) the adhesion was found to be good. However, with the LS-EC-CS substrates, one out of the three (marked in red circle) tests failed, wherein the entire CS layer (along with the printed Ag) got exfoliated as-is off of the EC surface, indicating an adhesion failure.

Physiological testing:

In terms of resistance to biologically relevant environments, no changes in the consistency of the substrates are observed within mammalian physiological temperature ranges and the substrate remains unaffected under constant submergence in Phosphate Buffer Saline (PBS) for over 30 days. The results do not vary when the fluid environment is changed to 10 M of NaCl solution for 30 days.

Additionally, to test for cytocompatibility, we exposed the substrates to sugar-water containing yeast cells (Saccharomyces cerevisiae) kept at 37°C and observed the growth of yeast cells on the substrate surface in a sealed environment. Yeast growth rendered the substrate milky white in color and the proliferation could be maintained for a month before being stopped (**Figure S13**).

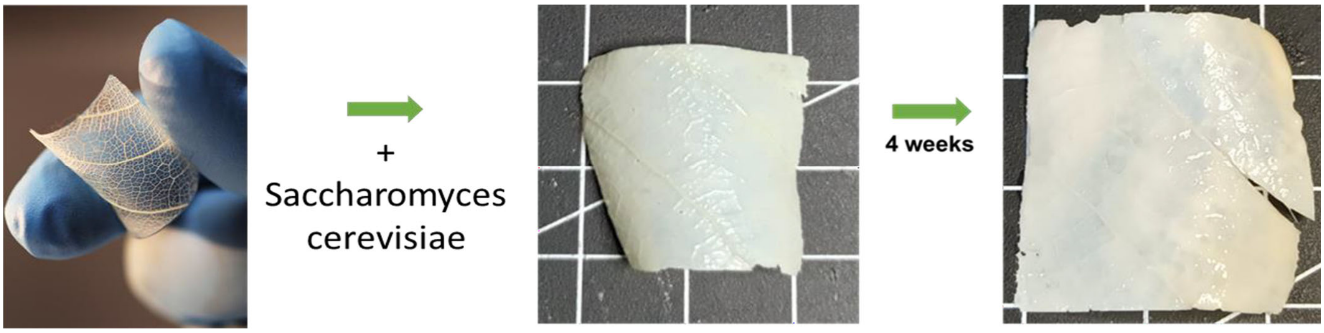

Figure S13: Yeast cell growth on LS-EC substrates. LS-EC substrates can sustain yeast cell colonies for over 4 weeks (right) before the experiments were stopped.

A

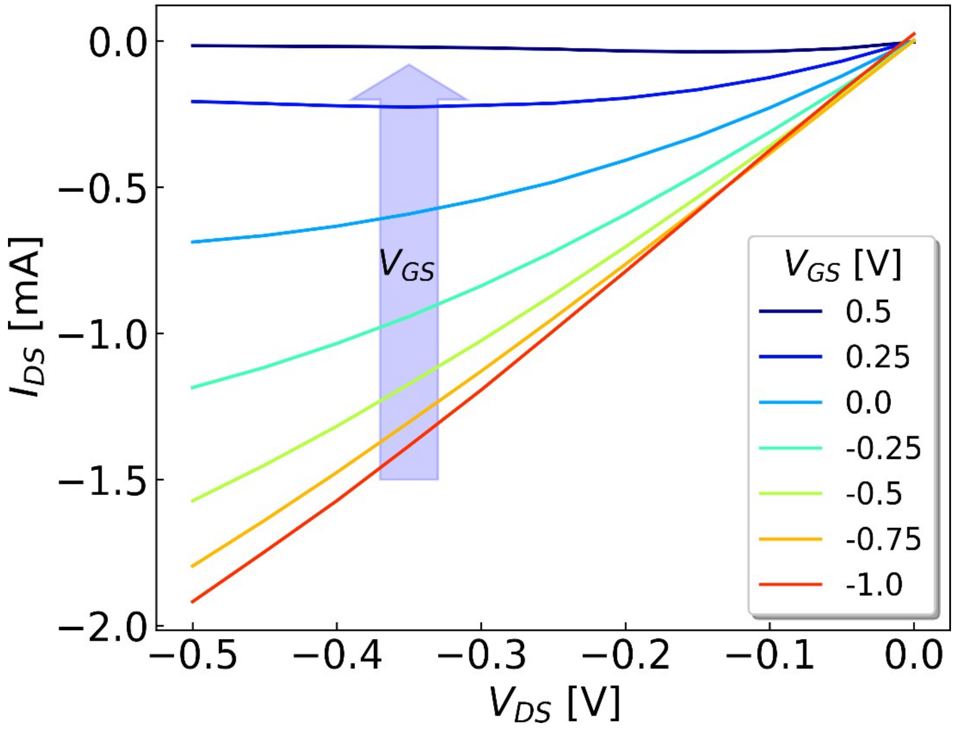

B

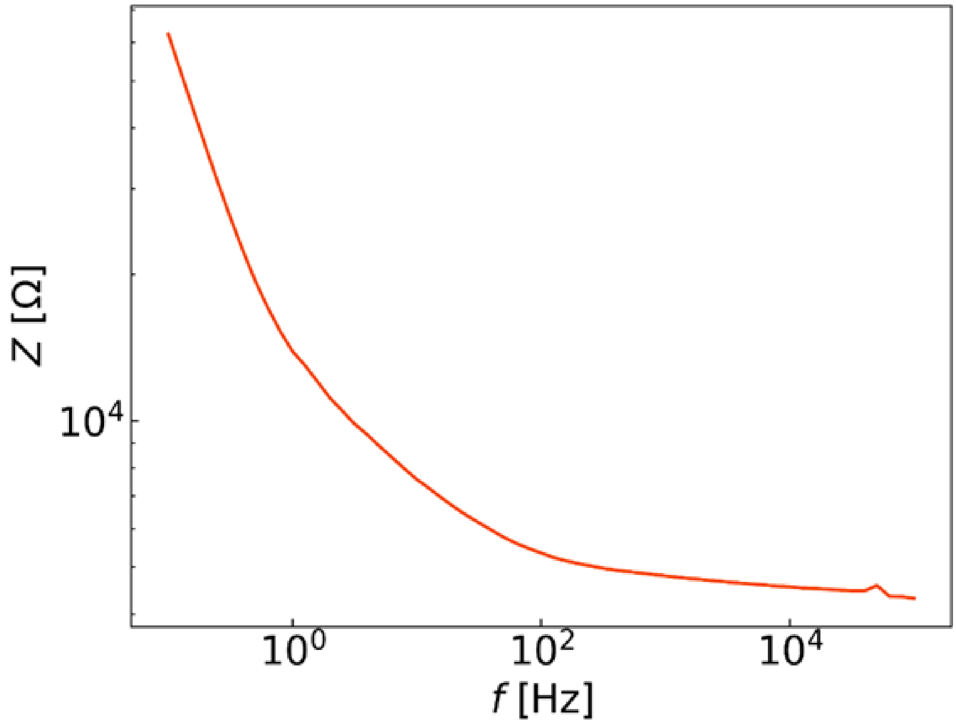

Figure S141: Further measurements of OECTs fabricated on the LS-EC substrates (A) Output characteristics of the inkjet-printed OECT (B) Impedance characteristics of the OECT

A

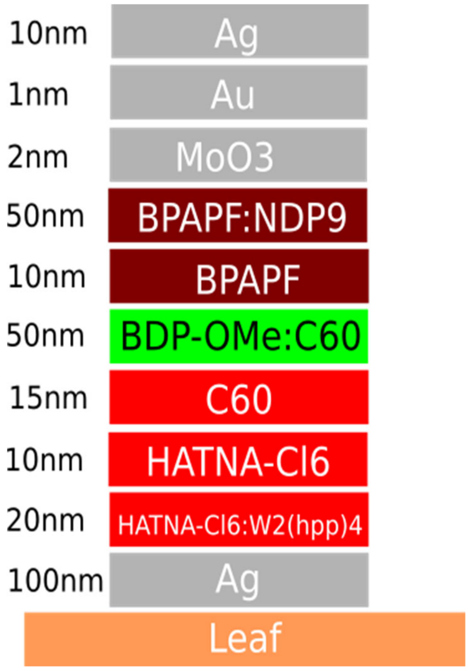

B

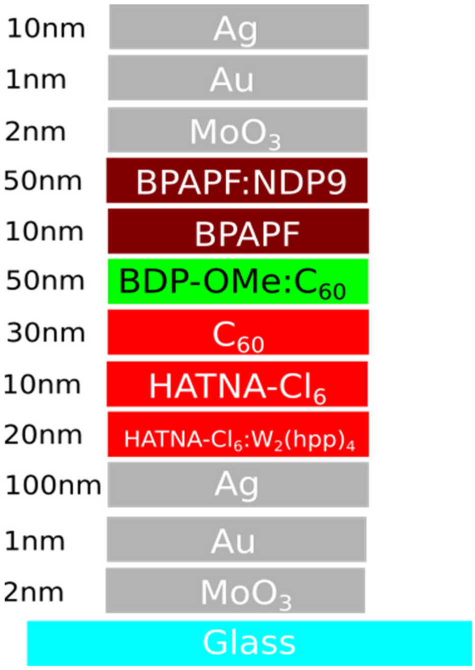

C

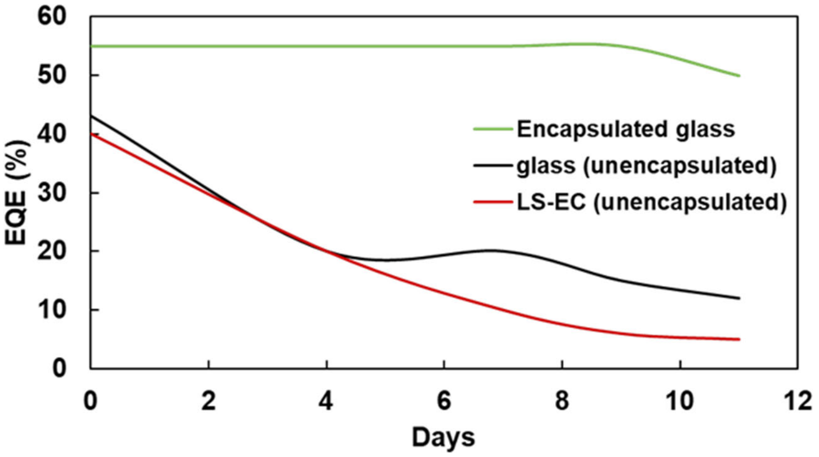

Figure S15: OPD fabrication and stability. OPD stacks (A) On LS-EC substrate (B) On glass (C) Deterioration of EQE over time for standard encapsulated devices (green, top), unencapsulated devices on glass (black, middle), and unencapsulated devices on LS-EC substrates (bottom, red).

Note: Devices built on LS-EC are taken without encapsulation, while devices built on glass are encapsulated. Hence, the EQE differs below 400nm.

Movie S1. - MCU based circuit on leaf-substrate (https://datashare.tu-dresden.de/s/tezdDFZaoK5G87J)

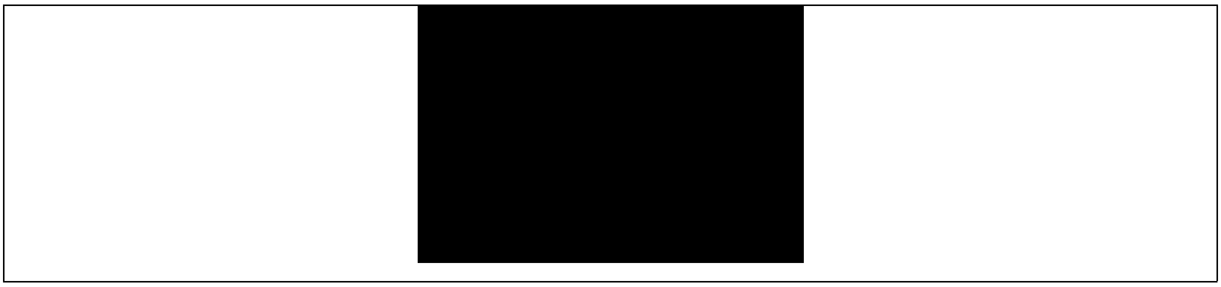

Movie S2. - Components shown in Video-1 extracted and recycled to replicate the circuit on a fresh leaf substrate (https://datashare.tu-dresden.de/s/EDwH6NsjsyZgm3L)

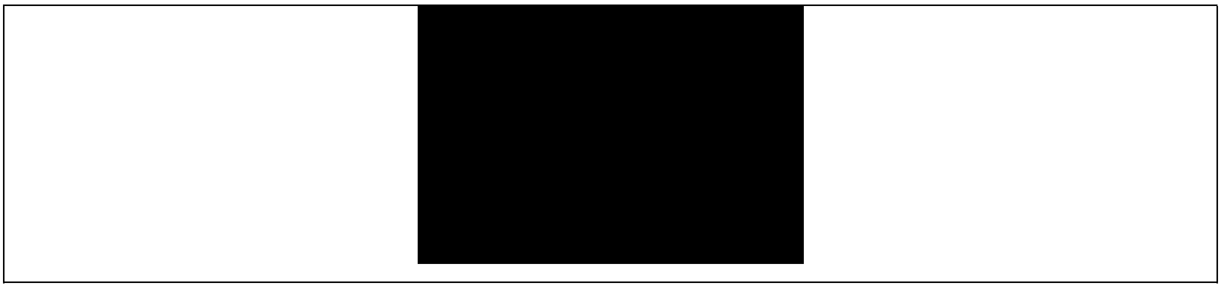

Benchmarking

For a clear assessment of the advantages garnered from implementing Leaftronics, it is crucial to compare its properties with other established technologies for substrates. As the most relevant property, we first determined

the kilogram of carbon dioxide equivalent per kilogram ($kgCO_2e/kg$) figure of merit for our substrates as shown in Table S3. In one leaf substrate fabrication process, we can produce up to 200 leaf substrates with a size of 2.5 x 2.5cm^2, which refers to a mass of 100g of green leaves. This amount corresponds to a substrate area of 1.25m^2. The following analysis is based on our lab-scale process and hence, further reduction of CO_2 emissions based on scale and process optimization are expected. Furthermore, the material consumption is provided with a large safety margin (e.g., for substrate mass and chitosan in Table S3) in order to have a conservative estimation of the overall CO_2 emission.

Table S3: Summary of the leaf substrate fabrication process (lab-scale) and analysis of $kgCO_2e/100g$ of leaf substrates.

S. No.	Material	Quantity used	$kgCO_2e/100g$ substrates	Remarks
1	Leaf	2.5 x 2.5cm^2 (100g)	~ 0.01 [101]	Leaf based carbon footprints are primarily calculated for store bought products which are packaged for long term storage which contributes 50% of the emission footprint. This value is eschewed here since fresh leaves can be directly harvested.
2	H_2O (tap water)	1L	0.1 [102]	Published values range between 0 and 0.1/kg, max. assumed
3	$Na_2CO_3.10H_2O$	100g	0.046 [103]	
4	Heating @ 120°C	4 hours	0.15	Assuming 100W power consumption with a German electricity carbon footprint of 385 gCO_2e/kWh [104]
5	ultrasonication	1 hour	0.008	Assuming 20W power consumption with a German electricity carbon footprint of 385 gCO_2e/kWh [104]
6	Ethyl cellulose<br>Industrial synthesis - heating alkali cellulose with ethyl chloride			Direct value for Ethyl cellulose not available: we approximated the effective value based on its industrial manufacturing process [105] below:

	(etherification) [106] [107]			
	Ethyl chloride *(kgCO_2e/kg = 1.5)* [107]		0.001	A leaf substrate weighs approximately 150mg. To keep calculations conservative, we use a mass of 500mg/substrate. Assuming 70% (350mg) of it is made up of EC, then based on the chemical reaction, approximately 50% (175mg) of the alkali cellulose used for synthesis (before it is reacted with ethyl chloride gas) will be pure cellulose, which, based on its per kg emission value will result in 0.00058 kgCO_2e/175mg of emissions. Since the reported per kg emission values of both Ethyl chloride and NaOH are much lower than cellulose already [107] [109], we can safely assume that these will be lower at all scales. Incorporating wastage and sub-par reaction efficiency, we assume the same emission values for ethyl chloride and NaOH as we did for cellulose in order to keep the estimation on the higher end.
	cellulose film *(kgCO_2e/kg = 3.3)* [108]		0.001	
	NaOH *(kgCO_2e/kg = 0.63)* [109]		0.001	
8	Solvent		0.09 [110]	Although 2-Butoxyethanol was used in this work, ethyl cellulose also dissolves readily in ethanol, which is also an organic solvent with similar properties.
7	Curing at 120°C after dip-coating and placement on chitosan coated glass	4 hours	0.15	Assuming 100W power consumption with a German electricity carbon footprint of 385 gCO_2e/kWh [104]
8	Chitosan (kgCO_2e/kg = 59.22) [111]	25g	1.5	Assuming that the thin coating of chitosan contributes about 5% of the mass of a substrate, the effective mass of chitosan for 100g would be 5g. However, due to the nature of the spin-coating process, we multiply this figure by 5 to account for losses.

**Total Carbon footprint (TCF) for Leaf substrate fabrication:**	**~ 2 $kgCO_2e$/100 g**	**100g of substrate will have approximately 1.25 m^2 area. Hence, for 1 m^2 area TCF = ~ 1.64 $kgCO_2e/m^2$**

The current ubiquitously employed substrate for PCB manufacturing is the glass-epoxy based FR4 board. However, in the past decades, paper has been the most widely recommended bio-derived substrate to replace FR4 [112] [113] [114], especially for single layer, high volume, and short lifespan circuitry [112] [115]. Having approximated the carbon footprint of our substrates, we also compare their properties in Table 2 (main text) with FR4 boards and paper in order to achieve a benchmark for commercial PCB fabrication.
Table 2 in the main text shows that the leaf substrates create less than half the carbon dioxide emission during fabrication compared to paper and almost ten times less than the industry standard. Additionally, further coatings are often required in order to make paper suitable for commercial PCB fabrication [112] which is not the case with Leaftronic substrates. An additional coating on paper also increases its overall carbon footprint and can reduce its degree of compostability while exacerbating the cracking of conducting tracks due to differences in thermal expansion during soldering [116]. In addition to these important TCF considerations, we highlight another property of the Leaftronic substrates i.e., their ability to withstand reflow soldering temperature profiles of >200°C, thus enabling the use of commercially viable soldering processes (as demonstrated in Figure 3A.2 in the main text). Such a process is difficult to implement on paper-based PCBs, which limits their implementation on a large scale by the PCB industry.

The use of additive metallization processes such as printing offers an enormous potential for the reduction of the TCF during PCB fabrication independently of the choice of the substrate. However, even though the amount of metal used in ink for additive manufacturing is low, the use of Ag can still contribute a significantly large carbon footprint of about 56 kg of CO_2 equivalent, which is about 14 times more than that of Cu based conductors on commercial PCBs [117]. Therefore, there is a profound need to introduce methodologies for extracting Ag from PCBs made using printed electronics. Ag extraction from paper substrates without damaging them is a major challenge since the ink absorbs into the fibres [117], however, with Leaftronic substrates, the Ag can be extracted via an undemanding room-temperature ultrasonication process carried out in mildly acidic conditions (pH<6) (main text section titled - Recycling). This method not only separates the Ag interconnects but also does not damage the substrate which can be recoated with chitosan (to make subsequent Ag extraction just as facile) before being upcycled into a fresh PCB. The separated Ag and the electronic components (**Figure S16**) can be filtered out of the solution since the micro-particle ink we developed for this work is released from

the surface as large, agglomerated chunks. Life cycle assessments for printed electronics have shown that the net carbon footprint of biodegradable substrates like paper can be reduced by a factor of 5 by simply introducing Ag recovery into the system [117]. However, this recovery process is difficult due to the penetration of the ink into the cellulose fibres. In such a sense, the TCF of Leaftronics can be much lower once the environmental benefits gained by Ag recovery are accounted for. This is especially significant since Leaftronic substrates are specifically suited for low carbon footprint recovery of components and Ag interconnects.

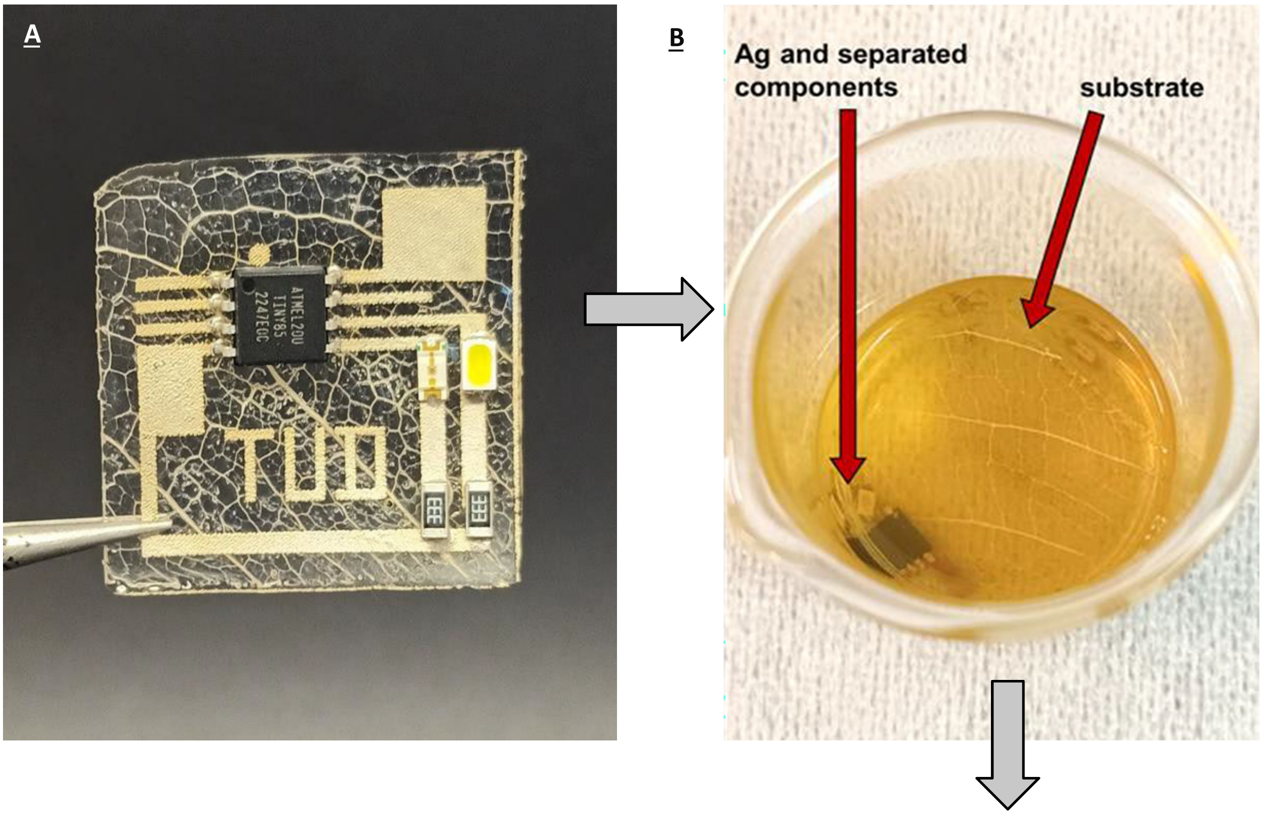

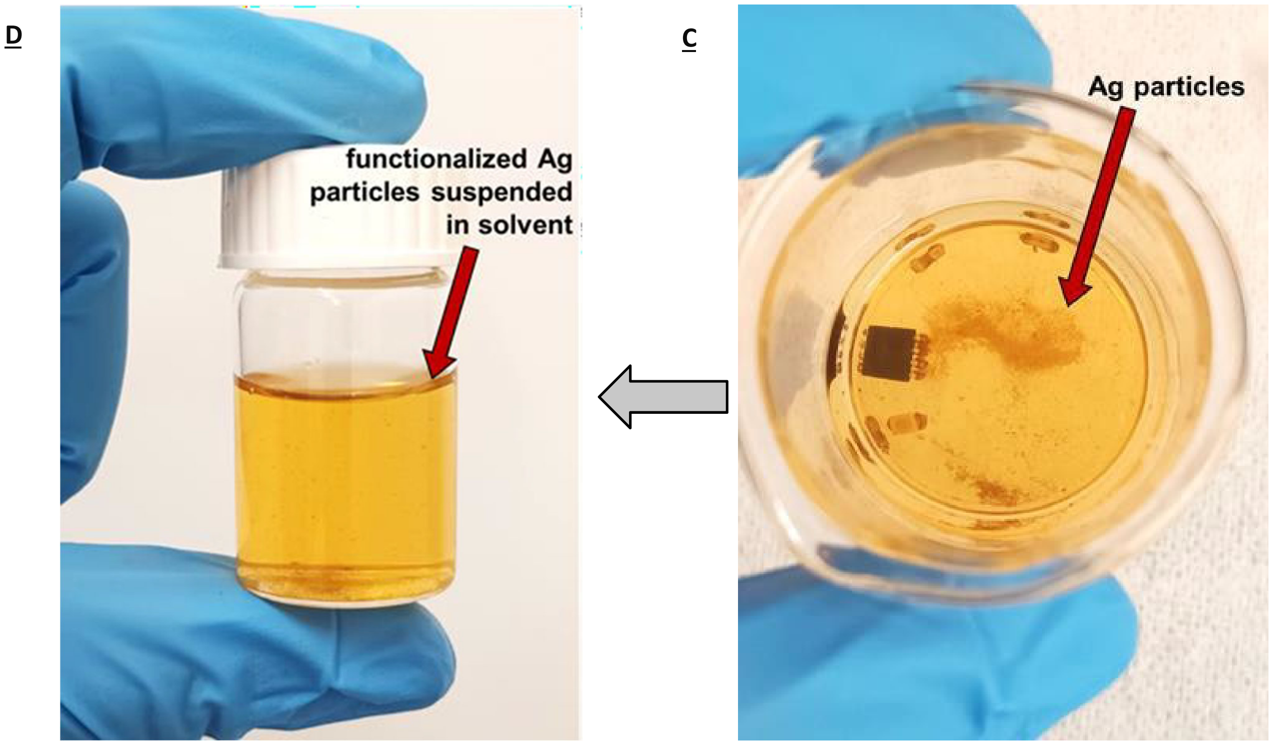

Figure S16: Upcycling of printed Ag and electronic components (A) Leaftronic PCB (B) Components and Ag interconnects extracted via ultrasonication under mildly acidic conditions (C) Ag particles and components settled at the bottom of the vessel (D) Polyethyleneimine (PEI) functionalized Ag particles in 2 Butoxyethanol as upcycled supplements to be added to Ag ink during the PCB fabrication step.

T_g measurements

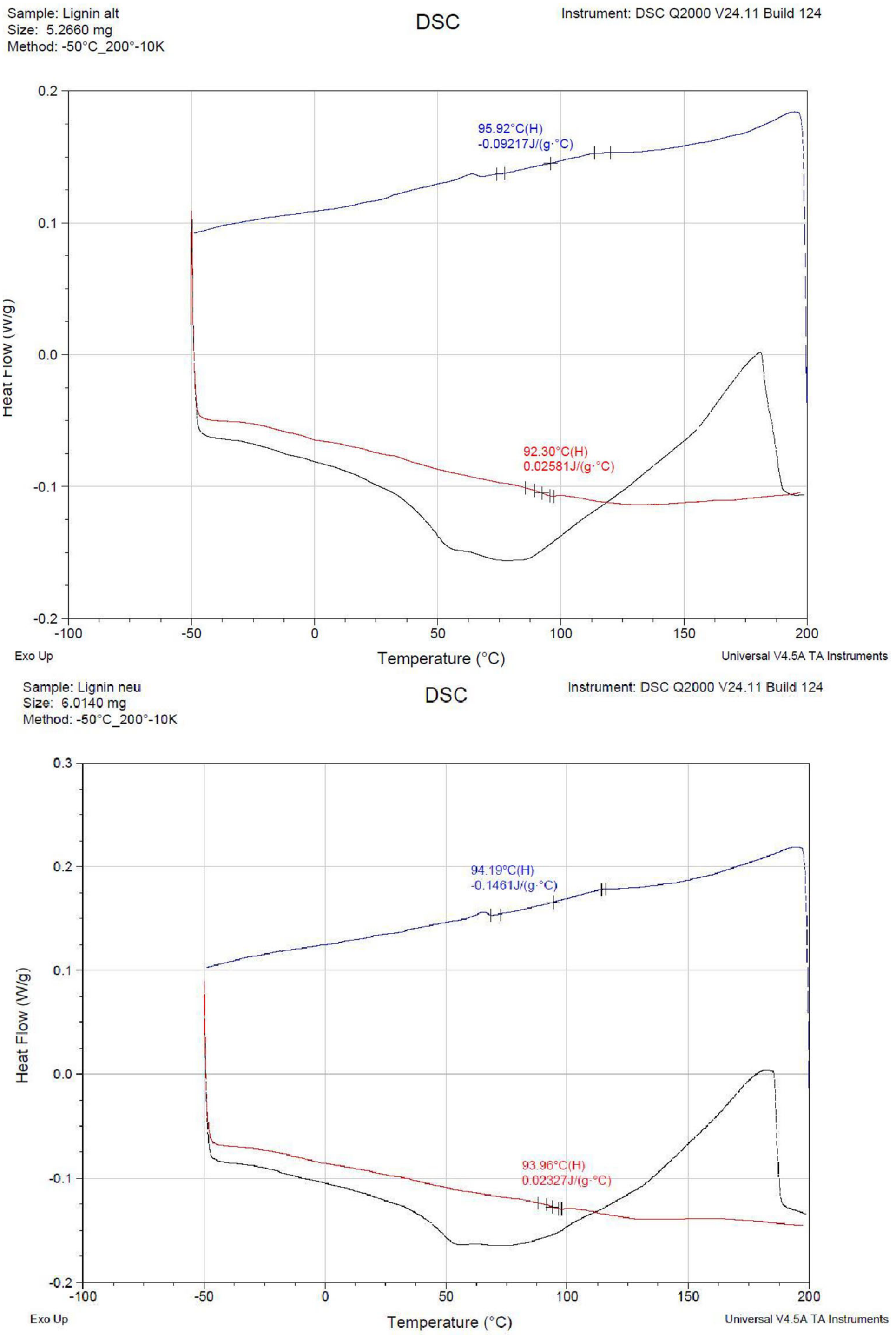

Figure S17: Glass transition measurement of LS-EC substrates. Results of T_g measurements performed using Differential scanning calorimetry (DSC). The first heating process is the black curve (bottom), the cooling process is shown in blue (top) and the second heating process is the red curve (middle).

Future Perspective:

The potential of the Leaftronic platform is evident in terms of the research results, however the techno-economic impact could only be studied at the laboratory scale for now due to the novelty of the system. Further research is needed to more accurately benchmark the environmental advantage garnered via the adoption of Leaftronics. However, the initial results are extremely promising due to the possibility of metal recovery and the low TCF of the substrates.

A step further from the analysis of the TCF is the Lifecycle assessment (LCA) which quantifies how a product would impact the environment during its entire lifecycle. It is majorly performed by understanding the goals and scope of the analysis, performing a lifecycle inventory (LCI), assessing the impact of the lifecycle, and finally interpreting the acquired data as per the ISO 14044 standard. Here we have assessed the leaf substrate within the scope of a general-purpose PCB and have focused on its CO_2 emissions during production as a part of its LCI. A Life Cycle Assessment (LCA) in terms of processes involved and the materials employed can further validate the outstanding potential that Leaftronics holds for the global electronics industry in terms of significantly reducing the carbon footprint, while conforming to the existing manufacturing process chains. The LCA system boundaries are shown in **Figure S18**. We consider the sourcing of raw leaves, the extraction of the lignocellulose skeletons, substrate formation, PCB fabrication and assembly, recovery of Ag interconnects and electronic components before finally considering substrate reuse/disposal.

As **Figure S18** demonstrates, the Waste from Electrical and Electronic Equipment (WEEE) considerations when made within the system boundaries provide multiple positive feedback options that reinforce different sections of the process chain. This reinforcement not only provides further reduction of the TCF but also improves the process costs which should scale up with production volume. This publication already demonstrates the possibility of upcycling extracted components back into the PCB assembly stage (Fig. 3A of the main text) along with the upcycling of the extracted Ag as a supplementing addition to conducting ink used during the PCB fabrication stage (**Figure S16**). Both these processes when implemented will significantly contribute to a further reduction in the CO_2 footprint of Leaftronic systems.

Finally, the LCA also shows benefits at the end-of-life period, i.e., at the point where the materials are beyond feasible reuse. The PCB, being completely biodegradable (Fig. 3C main text), can be easily composted in aerobic conditions to return nutrients to the environment as fertilizers. The Ag extracted before this step still gets upcycled into inks. Additionally, there is also a possibility of placing the entire Leaftronics system into dedicated biogas generation facilities where methane produced from anaerobic degradation can be used as an

energy input at the system boundary (**Figure S18** top). The electronic components and metal leftovers can be extracted for recycling and up-cycling purposes at fixed intervals to conform with circular economy principles.

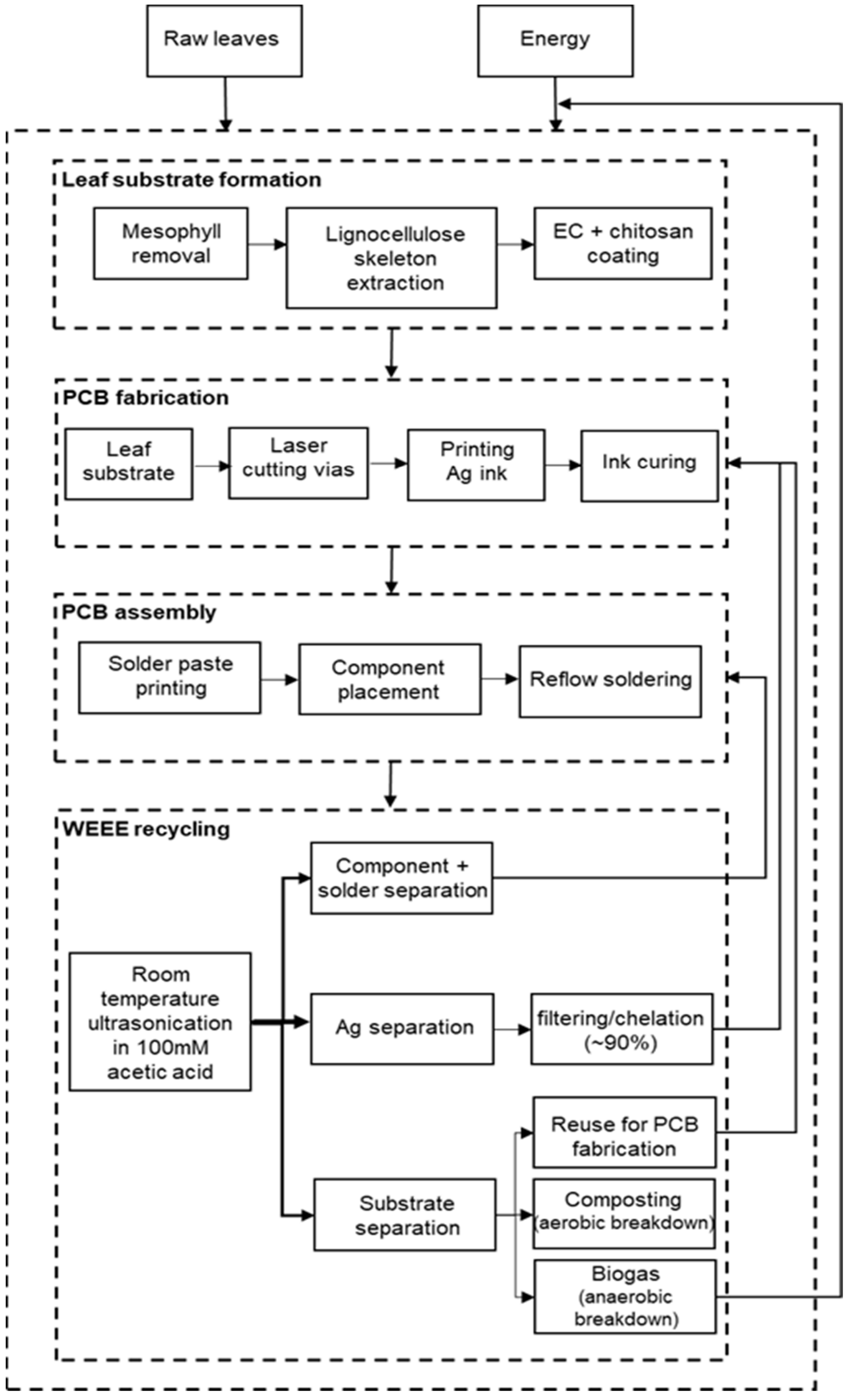

Figure S18: Life Cycle Assessment (LCA). System boundaries for cradle to grave life cycle when the substrates are implemented within the conventional electronics manufacturing process flow.

REFERENCES AND NOTES

1. V. Forti, C. P. Baldé, R. Kuehr, G. Bel, *The Global E-waste Monitor 2020: Quantities, Flows and the Circular Economy Potential* (United Nations University/United Nations Institute for Training and Research, SCYCLE Programme, International Telecommunication Union, and International Solid Waste Association, 2020).

2. F. P. Silvas, M. M. J. Correa, M. P. Caldas, V. T. de Moraes, D. C. R. Espinosa, J. A. S. Tenório, Printed circuit board recycling: Physical processing and copper extraction by selective leaching. *Waste Manag.* **46**, 503–510 (2015).

3. L. H. Yamane, V. T. de Moraes, D. C. R. Espinosa, J. A. S. Tenório, Recycling of WEEE: Characterization of spent printed circuit boards from mobile phones and computers. *Waste Manag.* **31**, 2553–2558 (2011).

4. S. Mühl, B. Beyer, Bio-organic electronics—Overview and prospects for the future. *Electronics* **3**, 444–461 (2014).

5. M. Irimia-Vladu, E. D. Głowacki, G. Voss, S. Bauer, N. S. Sariciftci, Green and biodegradable electronics. *Mater. Today* **15**, 340–346 (2012).

6. J. Kundu, F. Pati, Y. H. Jeong, D.-W. Cho, "Biomaterials for biofabrication of 3D tissue scaffolds" in *Biofabrication* (William Andrew Publishing, 2013), pp. 23–46.

7. W. G. Glasser, About making lignin great again—Some lessons from the past. *Front. Chem.* **7**, 565 (2019).

8. Z. Chen, T. Aziz, H. Sun, A. Ullah, A. Ali, L. Cheng, R. Ullah, F. U. Khan, Advances and applications of cellulose bio-composites in biodegradable materials. *J. Polym. Environ.* **31**, 2273–2284 (2023).

9. Y. Zhou, Y. Hu, Z. Tan, T. Zhou, Cellulose extraction from rice straw waste for biodegradable ethyl cellulose films preparation using green chemical technology. *J. Clean. Prod.* **439**, 140839 (2024).

10. N. B. Erdal, M. Hakkarainen, Degradation of cellulose derivatives in laboratory, man-made, and natural environments. *Biomacromolecules* **23**, 2713–2729 (2022).

11. Z. Jiang, T. Ngai, Recent advances in chemically modified cellulose and its derivatives for food packaging applications: A review. *Polymers* **14**, 1533 (2022).

12. M. Eck, S. T. Schwab, T. F. Nelson, K. Wurst, S. Iberl, D. Schleheck, C. Link, G. Battagliarin, S. Mecking, Biodegradable high-density polyethylene-like material. *Angew. Chem. Int. Ed. Engl.* **62**, e202213438 (2023).

13. N. T. Lotto, M. R. Calil, C. G. F. Guedes, D. S. Rosa, The effect of temperature on the biodegradation test. *Mater. Sci. Eng. C* **24**, 659–662 (2004).

14. Y. Lu, W. Yuan, Superhydrophobic/superoleophilic and reinforced ethyl cellulose sponges for oil/water separation: Synergistic strategies of cross-linking, carbon nanotube composite, and nanosilica modification. *ACS Appl. Mater. Interfaces* **9**, 29167–29176 (2017).

15. X. Xu, J. Li, X. Pan, X. Lin, Z. Li, Preparation of high tensile strength modified cellulose-based strippable coating and its application on radioactive decontamination at low-temperature. *Mater. Today Commun.* **34**, 105088 (2023).

16. D. W. Pearce, R. K. Turner, Economics of the natural resources and the environment. *Am. J. Agric. Econ.* **73**, 227–228 (1990).

17. A. Alcalde-Calonge, F. J. Sáez-Martínez, P. Ruiz-Palomino, Evolution of research on circular economy and related trends and topics. A thirteen-year review. *Ecol. Inform.* **70**, 101716 (2022).

18. M. N. Nassajfar, I. Deviatkin, V. Leminen, M. Horttanainen, Alternative materials for printed circuit board production: An environmental perspective. *Sustainability* **13**, 12126 (2021).

19. C. Lu, Y. Shen, X. Wang, S. Xu, J. Wang, Q. Yong, F. Chu, Biomimetic ultra-strong, ultra-tough, degradable cellulose-based composites for multi-stimuli responsive shape memory. *Int. J. Biol. Macromol.* **226**, 1468–1476 (2023).

20. D. Parker, Y. Daguerre, G. Dufil, D. Mantione, E. Solano, E. Cloutet, G. Hadziioannou, T. Näsholm, M. Berggren, E. Pavlopoulou, E. Stavrinidou, Biohybrid plants with electronic roots via in vivo polymerization of conjugated oligomers. *Mater. Horiz.* **8**, 3295–3305 (2021).

21. M. Davidovich-Pinhas, S. Barbut, A. G. Marangoni, Physical structure and thermal behavior of ethylcellulose. *Cellulose* **21**, 3243–3255 (2014).

22. K. Wasilewska, K. Winnicka, Ethylcellulose–A pharmaceutical excipient with multidirectional application in drug dosage forms development. *Materials* **12**, 3386 (2019).

23. V. Bampidis, V. Bampidis, G. Azimonti, M. de Lourdes Bastos, H. Christensen, B. Dusemund, M. Durjava, M. Kouba, M. López-Alonso, S. L. Puente, F. Marcon, B. Mayo, A. Pechová, M. Petkova, F. Ramos, R. E. Villa, R. Woutersen, M. Anguita, J. Galobart, P. Manini, M. V. Vettori, M. Innocenti, Safety and efficacy of ethyl cellulose for all animal species. *EFSA J.* **18**, 14 (2020).

24. R. W. Dickey, W. W. Dickhoff, "Dispersants and Seafood Safety Assessment of the potential impact of COREXIT oil dispersants on seafood safety. A White Paper for the Coastal Response Research Center. Dispersant Initiative and Workshop "The Future of Dispersant Use in Spill Response"" (Coastal Response Research Center, 2011).

25. J. Ge, M. Li, J. Fan, C. Celia, Y. Xie, Q. Chang, X. Deng, Synthesis, characterization, and antibacterial activity of chitosan-chelated silver nanoparticles. *J. Biomater. Sci. Polym. Ed.* **35**, 45–62 (2024).

26. Z. Li, J. Ma, R. Li, X. Yin, W. Dong, C. Pan, Fabrication of a blood compatible composite membrane from chitosan nanoparticles, ethyl cellulose and bacterial cellulose sulfate. *RSC Adv.* **8**, 31322–31330 (2018).

27. P. Shi, Y. Li, L. Zhang, Fabrication and property of chitosan film carrying ethyl cellulose microspheres. *Carbohydr. Polym.* **72**, 490–499 (2008).

28. A. Anand, M. M. Islam, R. Meitzner, U. S. Schubert, H. Hoppe, Introduction of a novel figure of merit for the assessment of transparent conductive electrodes in photovoltaics: Exact and approximate form. *Adv. Energy Mater.* **11**, 2100875 (2021).

29. M. A. Mohd Asri, N. A. Ramli, A. N. Nordin, Electrical performance and reliability assessment of silver inkjet printed circuits on flexible substrates. *J. Mater. Sci. Mater. Electron.* **32**, 16024–16037 (2021).

30. A. Claypole, J. Claypole, L. Kilduff, D. Gethin, T. Claypole, Stretchable carbon and silver inks for wearable applications. *Nanomaterials* **11**, 1200 (2021).

31. M. Kayaharman, H. Argasinski, J. Atkinson, K. Zhang, Y. N. Zhou, I. A. Goldthorpe, Enhancing and understanding the high stretchability of printable, conductive silver nanowire ink. *J. Electron. Mater.* **54**, 4634–4643 (2023).

32. M. Davidovich-Pinhas, S. Barbut, A. G. Marangoni, The gelation of oil using ethyl cellulose. *Carbohydr. Polym.* **117**, 869–878 (2015).

33. Merck KGaA, "Ethyl cellulose," Sigma-Aldrich; www.sigmaaldrich.com/DE/en/product/aldrich/200689 [accessed 26 July 2024].

34. P. V. Kozlov, G. I. Burdygina, The structure and properties of solid gelatin and the principles of their modification. *Polymer* **24**, 651–666 (1983).

35. L. Mehra, S. Mehra, N. Tiwari, T. Singh, H. Rawat, S. Belagavi, A. Jaimini, G. Mittal, Fabrication, characterization and evaluation of the efficacy of gelatin/hyaluronic acid microporous scaffolds suffused with aloe-vera in a rat burn model. *J. Biomater. Appl.* **36**, 1346–1358 (2022).

36. S. Nandy, S. Goswami, A. Marques, D. Gaspar, P. Grey, I. Cunha, D. Nunes, A. Pimentel, R. Igreja, P. Barquinha, L. Pereira, E. Fortunato, R. Martins, Cellulose: A contribution for the zero e-waste challenge. *Adv. Mater. Technol.* **6**, 2000994 (2021).

37. R. R. Nair, Organic electrochemical transistor on paper for the detection of halide anions in biological analytes. *Flex. Print. Electron.* **5**, 045004 (2020).

38. J. Liu, C. Yang, H. Wu, Z. Lin, Z. Zhang, R. Wang, B. Li, F. Kang, L. Shi, C. P. Wong, Future paper based printed circuit boards for green electronics: Fabrication and life cycle assessment. *Energy Environ. Sci.* **7**, 3674–3682 (2014).

39. M. Sun, Y. Wang, L. Shi, J. J. Klemeš, Uncovering energy use, carbon emissions and environmental burdens of pulp and paper industry: A systematic review and meta-analysis. *Renew. Sustain. Energy Rev.* **92**, 823–833 (2018).

40. E.-H. Song, J. Shang, D. M. Ratner, "9.08 - Polysaccharides" in *Polymer Science: A Comprehensive Reference* (Elsevier, 2012), pp. 137–155.

41. B.-H. Jun, Silver nano/microparticles: Modification and applications. *Int. J. Mol. Sci.* **20**, 2609 (2019).

42. M. A. Diab, A. Z. El-Sonbati, D. M. Bader, Thermal stability and degradation of chitosan modified by benzophenone. *Spectrochim. Acta A Mol. Biomol. Spectrosc.* **79**, 1057–1062 (2011).

43. S. Cheng, C.-M. Huang, M. Pecht, A review of lead-free solders for electronics applications. *Microelectron. Reliab.* **75**, 77–95 (2017).

44. Everlight Electronics Co. Ltd., Reverse Package Chip LED with Inner Lens (Technical Data Sheet 25-21/T1D-ANQHY/2A, 2006); www.reichelt.de/de/en/smd-led-1206-reverse-white-112-mcd-70--evl-25-21-t1d-a-p232086.html and https://cdn-reichelt.de/documents/datenblatt/A500/25-21-T1D-ANQHY-2A_ENG_TDS.pdf [accessed 31 July 2024].

45. A. Weissbach, L. M. Bongartz, M. Cucchi, H. Tseng, K. Leo, H. Kleemann, Photopatternable solid electrolyte for integrable organic electrochemical transistors: Operation and hysteresis. *J. Mater. Chem. C* **10**, 2656–2662 (2022).

46. M. Cucchi, H. Kleemann, H. Tseng, G. Ciccone, A. Lee, D. Pohl, K. Leo, Directed growth of dendritic polymer networks for organic electrochemical transistors and artificial synapses. *Adv. Electron. Mater.* **0**, 2100586 (2021).

47. B. Zareeipolgardani, A. Piednoir, B. Joyard-Pitiot, G. Depres, L. Charlet, J. Colombani, Multiscale investigation of the fate of silver during printed paper electronics recycling. *Composite Inter.* **30**, 671–684 (2023).

48. J. Wiklund, A. Karakoç, T. Palko, H. Yiğitler, K. Ruttik, R. Jäntti, J. Paltakari, A review on printed electronics: Fabrication methods, inks, substrates, applications and environmental impacts. *J. Manuf. Mater. Process.* **5**, 89 (2021).

49. L. F. Zemljič, Z. Peršin, P. Stenius, Improvement of chitosan adsorption onto cellulosic fabrics by plasma treatment. *Biomacromolecules* **10**, 1181–1187 (2009).

50. S. Z. Rogovina, Biodegradable polymer composites based on synthetic and natural polymers of various classes. *Polym. Sci. Ser. C* **58**, 62–73 (2016).

51. P. Shi, Q. Miao, Ü. Niinemets, M. Liu, Y. Li, K. Yu, K. J. Niklas, Scaling relationships of leaf vein and areole traits versus leaf size for nine Magnoliaceae species differing in venation density. *Am. J. Bot.* **109**, 899–909 (2022).

52. P. Keller, H. Kawasaki, Conductive leaf vein networks produced via Ag nanoparticle self-assembly for potential applications of flexible sensor. *Mater. Lett.* **284**, 128937 (2021).

53. V. Sharma, K. Jääskö, K. Yiannaco, A. Koivikko, V. Lampinen, V. Sariola, Performance comparison of fast, transparent, and biotic heaters based on leaf skeletons. *Adv. Eng. Mater.* **24**, 2101625 (2022).

54. H. Li, Y. Ma, Y. Huang, Material innovation and mechanics design for substrates and encapsulation of flexible electronics: A review. *Mater. Horiz.* **8**, 383–400 (2021).

55. X. Li, R. Li, Z. Liu, X. Gao, S. Long, G. Zhang, Integrated functional high-strength hydrogels with metal-coordination complexes and h-bonding dual physically cross-linked networks. *Macromol. Rapid Commun.* **39**, e1800400 (2018).

56. B. Ding, P. Zeng, Z. Huang, L. Dai, T. Lan, H. Xu, Y. Pan, Y. Luo, Q. Yu, H.-M. Cheng, B. Liu, A 2D material–based transparent hydrogel with engineerable interference colours. *Nat. Commun.* **13**, 1212 (2022).

57. L. Shi, R. Yang, S. Lu, K. Jia, C. Xiao, T. Lu, T. Wang, W. Wei, H. Tan, S. Ding, Dielectric gels with ultra-high dielectric constant, low elastic modulus, and excellent transparency. *NPG Asia Mater.* **10**, 821–826 (2018).

58. M. Kheirabadi, R. Bagheri, K. Kabiri, Swelling and mechanical behavior of nanoclay reinforced hydrogel: Single network vs. full interpenetrating polymer network. *Polym. Bull.* **72**, 1663–1681 (2015).

59. Y. Cheng, R. Wang, H. Zhaia, J. Sun, Stretchable electronic skin based on silver nanowire composite fiber electrodes for sensing pressure, proximity, and multidirectional strain. *Nanoscale* **9**, 3834–3842 (2017).

60. H. Niu, H. Wang, H. Zhoua, T. Lin, Ultrafine PDMS fibers: Preparation from in situ curing-electrospinning and mechanical characterization. *RSC Adv.* **4**, 11782–11787 (2014).

61. C. T. Pan, Y. C. Chen, P.-H. Lin, C. C. Hsieh, F. T. Hsu, P.-H. Lin, C. M. Chang, J. H. Hsu, J. C. Huang, Lens of controllable optical field with thin film metallic glasses for UV-LEDs. *Opt. Express* **22**, 14411–14424 (2014).

62. P. Bodö, J.-E. Sundgren, Titanium deposition onto ion-bombarded and plasma-treated polydimethylsiloxane: Surface modification, interface and adhesio. *Thin Solid Films* **136**, 147–159 (1986).

63. S. G. Kirtania, A. W. Elger, M. R. Hasan, A. Wisniewska, K. Sekhar, T. Karacolak, P. K. Sekhar, Flexible antennas: A review. *Micromachines* **11**, 847 (2020).

64. K. Zalewski, Z. Chyłek, W. A. Trzciński, A review of polysiloxanes in terms of their application in explosives. *Polymers* **13**, 1080 (2021).

65. N. S. Gupta, K.-S. Lee, A. Labouriau, Tuning thermal and mechanical properties of polydimethylsiloxane with carbon fibers. *Polymers* **13**, 1141 (2021).

66. E. T. Filipov, K. Liu, T. Tachi, M. Schenk, G. H. Paulino, Bar and hinge models for scalable analysis of origami. *Int. J. Solids Struct.* **124**, 26–45 (2017).

67. J. Chen, E. Dörsam, D. Spiehl, A. H. Tehrani, J. Da, "Stress-strain behavior of paper affected by the actual contact area" in *Progress in Paper Physics Seminar 2016* (Technical University of Darmstadt, 2016), pp. 47–53.

68. A. Hovi, P. Forsström, M. Mõttus, M. Rautiainen, Evaluation of accuracy and practical applicability of methods for measuring leaf reflectance and transmittance spectra. *Remote Sens. (Basel)* **10**, 10 (2018).

69. S. Simula, S. Ikäläinen, K. Niskanen, T. Varpula, Measurement of the dielectric properties of paper. *J. Imaging Sci. Technol.* **43**, 472–477 (1999).

70. Y. Wang, T. Yang, J. Li, Glass transition temperature and mechanical properties in amorphous region of transformer insulation paper by molecular dynamic simulations. *IEEE Int. Symp. Electr. Insul.* , 164–168 (2012).

71. A. de Ruvo, R. Lundberg, S. Martin-Löf, C. Söremark, "Influence of temperature and humidity on the elastic and expansional properties of paper and the constituent fibre" in *Trans. of the Vth Fund. Res. Symp.* (Cambridge, 1973), pp. 785–806.

72. M. Kaltenbrunner, T. Sekitani, J. Reeder, T. Yokota, K. Kuribara, T. Tokuhara, M. Drack, R. Schwödiauer, I. Graz, S. Bauer-Gogonea, S. Bauer, T. Someya, An ultra-lightweight design for imperceptible plastic electronics. *Nature* **499**, 458–463 (2013).

73. C. Lechat, A. R. Bunsell, P. Davies, Tensile and creep behaviour of polyethylene terephthalate and polyethylene naphthalate fibres. *J. Mater. Sci.* **46**, 528–533 (2011).

74. Z. Li, S. K. Sinha, G. M. Treich, Y. Wang, Q. Yang, A. A. Deshmukh, G. A. Sotzing, Y. Cao, All-organic flexible fabric antenna for wearable electronics. *J. Mater. Chem. C* **8**, 5662–5667 (2020).

75. W. A. Macdonald, M. K. Looney, D. MacKerron, R. Eveson, R. Adam, K. Hashimoto, K. Rakos, Latest advances in substrates for flexible electronics. *J. Soc. Inf. Disp.* **15**, 1075–1083 (2007).

76. H.-S. Lee, J.-O. Bang, H.-J. Lee, G.-J. Lee, K.-H. Chai, S.-B. Jung, "Analysis of thermo-mechanical behavior of ITO layer on PET substrate" in *2011 IEEE 61st Electronic Components and Technology Conference (ECTC)* (IEEE, 2011), pp. 1796–1799.

77. R. Xu, J. W. Lee, T. Pan, S. Ma, J. Wang, J. H. Han, Y. Ma, J. A. Rogers, Y. Huang, Designing thin, ultrastretchable electronics with stacked circuits and elastomeric encapsulation materials. *Adv. Funct. Mater.* **27**, 1604545 (2017).

78. T. Uhrmann, L. Bär, T. Dimopoulos, N. Wiese, M. Rührig, A. Lechner, Magnetostrictive GMR sensor on flexible polyimide substrates. *J. Magn. Magn. Mater.* **307**, 209–211 (2006).

79. M. A. Riheen, T. Nguyen, T. K. Saha, T. Karacolak, P. K. Sekhar, CPW fed wideband bowtie slot antenna on PET substrate. *Prog. Electromagn. Res. C* **101**, 147–158 (2020).

80. G.-L. Jiang, D.-Y. Wang, H.-P. Du, X. Wu, Y. Zhang, Y.-Y. Tan, L. Wu, J.-G. Liu, A. X.-M. Zhang, Reduced coefficients of linear thermal expansion of colorless and transparent semi-alicyclic polyimide films via incorporation of rigid-rod amide moiety: Preparation and properties. *Polymers* **12**, 413 (2020).

81. K. A. Sierros, D. R. Cairns, J. S. Abell, S. N. Kukureka, Pulsed laser deposition of indium tin oxide films on flexible polyethylene naphthalate display substrates at room temperature. *Thin Solid Films* **518**, 2623–2627 (2010).

82. M.-C. Choi, Y. Kim, C.-S. Ha, Polymers for flexible displays: From material selection to device applications. *Prog. Polym. Sci.* **33**, 581–630 (2008).

83. A. Sudheshwar, N. Malinverno, R. Hischi, B. Nowack, C. Som, The need for design-for-recycling of paper-based printed electronics – A prospective comparison with printed circuit boards. *Resour. Conserv. Recycl.* **189**, 106757 (2023).

84. M. Gao, C. C. Shih, S. Y. Pan, C. C. Chueh, W. C. Chen, Advances and challenges of green materials for electronics and energy storage applications: From design to end-of-life recovery. *J. Mater. Chem. A* **6**, 20546–20563 (2018).

85. J. Spokes, Jiva Materials (2022); www.jivamaterials.com/technology/ [accessed 23 October 2023].

86. A. C. Dias, L. Arroja, Comparison of methodologies for estimating the carbon footprint – Case study of office paper. *J. Clean. Prod.* **24**, 30–35 (2012).

87. C. Mori de Oliveira, R. Bellopede, A. Tori, P. Marini, Study of metal recovery from printed circuit boards by physical-mechanical treatment processes. *Mater. Proc.* **5**, 121 (2021).

88. V. Premur, A. A. Vučinić, D. Vujević, G. Bedeković, The possibility for environmental friendly recycling of printed circuit boards. *J. Sustain. Devel. Energy Water Environ. Syst.* **4**, 14–22 (2016).

89. H. Andersson, J. Sidén, V. Skerved, X. Li, L. Gyllner, Soldering surface mount components onto inkjet printed conductors on paper substrate using industrial processes. *IEEE Trans. Compon. Packaging Manuf. Technol.* **6**, 478–485 (2016).

90. A. Pietrikova, T. Lenger, L. Livovsky, "Mechanical properties of sandwich electronic boards after multi reflow exposure" in 2020 *International Conference on Diagnostics in Electrical Engineering (Diagnostika)* (IEEE, 2020), pp. 1–4.

91. Y. Wang, T. Yang J. Li, "Glass transition temperature and mechanical properties in amorphous region of transformer insulation paper by molecular dynamic simulations" *in 2012 IEEE International Symposium on Electrical Insulation* (IEEE, 2012), pp. 164–168.

92. K. Scheuer, J. Holmes, E. Galyaev, D. Blyth, R. Alarcon, Radiation effects on FR4 printed circuit boards. *IEEE Trans. Nucl. Sci.* **67**, 1846–1851 (2020).

93. S. Simula, S. Ikäläinen, K. Niskanen, Measurement of the dielectric properties of paper. *J. Imaging Sci. Technol.* **43**, 157–160 (1999).

94. E. Rio, F. Boulogne, Withdrawing a solid from a bath: How much liquid is coated? *Adv. Colloid Interface Sci.* **247**, 100–114 (2017).

95. C. Gutfinger, J. A. Tallmadge, Films of nonnewtonian fluids adhering to flat plates. *AIChE J.* **11**, 403–413 (1965).

96. M. Fikry, M. Mohie, M. Gamal, A. Ibrahim, G. Genidy, Superior control for physical properties of sputter deposited ITO thin-films proper for some transparent solar applications. *Opt. Quant. Electron* **53**, 122 (2021).

97. C. P. Constantin, M. Aflori, R. F. Damian, R. D. Rusu, Biocompatibility of polyimides: A mini-review. *Materials* **12**, 3166 (2019).

98. N. K. Mondal, S. Kundu, P. Debnath, A. Mondal, K. Sen, Effects of polyethylene terephthalate microplastic on germination, biochemistry and phytotoxicity of Cicer arietinum L. and cytotoxicity study on Allium cepa L. *Environ. Toxicol. Pharmacol.* **94**, 103908 (2022).

99. D. A. van den Ende, R. Hendriks, R. Cauchois, W. A. Groen, Large area photonic flash soldering of thin chips on flex foils for flexible electronic systems: In situ temperature measurements and thermal modelling. *Electro. Mater. Lett.* **10**, 1175–1183 (2014).

100. V. Bampidis, G. Azimonti, M. de Lourdes Bastos, H. Christensen, B. Dusemund, M. Durjava, M. Kouba, M. López-Alonso, S. L. Puente, F. Marcon, B. Mayo, A. Pechová, M. Petkova, F. Ramos, R. E. Villa, R. Woutersen, M. Anguita, J. Galobart, P. Manini, M. V. Vettori, M. Innocenti, Scientific Opinion on the safety and efficacy of ethyl cellulose for all animal species. *EFSA J.* **18**, 6210 (2020).

101. CarbonCloud, "The climate intelligence platform"; https://apps.carboncloud.com/climatehub/product-reports/050700566887/USA [accessed 23 October 2023].

102. CarbonCloud, "The climate intelligence platform"; https://apps.carboncloud.com/climatehub/product-reports/id/5435817490 [accessed 23 October 2023].

103. CarbonCloud, "The climate intelligence platform"; https://apps.carboncloud.com/climatehub/product-reports/id/44158023257 [accessed 23 October 2023].

104. I. Tiseo, “Carbon intensity of the power sector in Germany from 2000 to 2022 (in grams of CO_2 per kilowatt-hour),” Statista; www.statista.com/statistics/1290224/carbon-intensity-power-sector-germany/ [accessed 23 October 2023].

105. T. Dürig, K. Karan, “Binders in wet granulation” in *Handbook of Pharmaceutical Wet Granulation* (Academic Press, 2019), pp. 317–349.

106. O. A. Adeleke, Premium ethylcellulose polymer based architectures at work in drug delivery. *Int. J. Pharm. X* **1**, 2590–1567 (2019).

107. A. H. Tullo, “The search for greener ethylene,” *Chemical & Engineering News (C&EN)*, 15 March 2021; https://cen.acs.org/business/petrochemicals/search-greener-ethylene/99/i9 [accessed 23 October 2023].

108. CarbonCloud, “The climate intelligence platform”; https://apps.carboncloud.com/climatehub/product-reports/id/1267952392638 [accessed 23 October 2023].

109. CarbonCloud, “The climate intelligence platform”; https://apps.carboncloud.com/climatehub/product-reports/id/183314951001 [accessed 23 October 2023].

110. CarbonCloud, “The climate intelligence platform”; https://apps.carboncloud.com/climatehub/product-reports/id/48954332807 [accessed 14 June 2024].

111. A. Riofrio, T. Alcivar, H. Baykara, Environmental and economic viability of chitosan production in Guayas-Ecuador: A robust investment and life cycle analysis. *ACS Omega* **6**, 23038–23051 (2021).

112. A. Sudheshwar, N. Malinverno, R. Hischi, B. Nowack, C. Som, The need for design-for-recycling of paper-based printed electronics – A prospective comparison with printed circuit boards. *Resour. Conserv. Recycl.* **189**, 106757 (2023).

113. H. Wu, S. W. Chiang, W. Lin, C. Yang, Z. Li, J. Liu, X. Cui, F. Kang, C. P. Wong, Towards practical application of paper based printed circuits: Capillarity effectively enhances conductivity of the thermoplastic electrically conductive adhesives. *Sci. Rep.* **4**, 6275 (2014).

114. J. Liu, C. Yang, H. Wu, Z. Lin, Z. Zhang, R. Wang, B. Li, F. Kang, L. Shi, C. P. Wong, Future paper based printed circuit boards for green electronics: Fabrication and life cycle assessment. *Energy Environ. Sci.* **7**, 3674–3682 (2014).

115. M. Keskinen, "End-of-life options for printed electronics" in *Waste Electrical and Electronic Equipment (WEEE) Handbook* (Woodhead Publishing, 2012), pp. 352–364.

116. H. Andersson, J. Sidén, V. Skerved, X. Li, L. Gyllner, Soldering surface mount components onto inkjet printed conductors on paper substrate using industrial processes. *IEEE Trans. Compon. Packaging Manuf. Technol.* **6**, 478–485 (2016).

117. B. Zareeipolgardani, A. Piednoir, B. Joyard-Pitiot, G. Depres, L. Charlet, J. Colombani, Multiscale investigation of the fate of silver during printed paper electronics recycling. *Compos. Inter.* **30**, 671–684 (2023).
